# Autophagy Is Involved in Mesenchymal Stem Cell Death in Coculture with Chondrocytes

**DOI:** 10.1177/1947603520941227

**Published:** 2020-07-22

**Authors:** Carlo Alberto Paggi, Amel Dudakovic, Yao Fu, Catalina Galeano Garces, Mario Hevesi, Daniela Galeano Garces, Allan B. Dietz, Andre J. van Wijnen, Marcel Karperien

**Affiliations:** 1 Department of Developmental BioEngineering, University of Twente, Enschede, Netherlands; 2 Department of Orthopedic Surgery, Mayo Clinic, Rochester, MN, USA; 3 Department of Biochemistry and Molecular Biology, Mayo Clinic, Rochester, MN, USA; 4 Department of Laboratory Medicine and Pathology, Mayo Clinic, Rochester, MN, USA

**Keywords:** autophagy, coculture, chondrocytes, mesenchymal stem cells, pellet

## Abstract

**Objective:**

Cartilage formation is stimulated in mixtures of chondrocytes and human adipose–derived mesenchymal stromal cells (MSCs) both *in vitro* and *in vivo*. During coculture, human MSCs perish. The goal of this study is to elucidate the mechanism by which adipose tissue–derived MSC cell death occurs in the presence of chondrocytes.

**Methods:**

Human primary chondrocytes were cocultured with human MSCs derived from 3 donors. The cells were cultured in monoculture or coculture (20% chondrocytes and 80% MSCs) in pellets (200,000 cells/pellet) for 7 days in chondrocyte proliferation media in hypoxia (2% O_2_). RNA sequencing was performed to assess for differences in gene expression between monocultures or coculture. Immune fluorescence assays were performed to determine the presence of caspase-3, LC3B, and P62.

**Results:**

RNA sequencing revealed significant upregulation of >90 genes in the 3 cocultures when compared with monocultures. STRING analysis showed interconnections between >50 of these genes. Remarkably, 75% of these genes play a role in cell death pathways such as apoptosis and autophagy. Immunofluorescence shows a clear upregulation of the autophagic machinery with no substantial activation of the apoptotic pathway.

**Conclusion:**

In cocultures of human MSCs with primary chondrocytes, autophagy is involved in the disappearance of MSCs. We propose that this sacrificial cell death may contribute to the trophic effects of MSCs on cartilage formation.

## Introduction

There is a range of current treatment modalities for symptomatic and focal cartilage defects.^1,2^ These include bone marrow stimulation techniques like microfracture or autologous chondrocyte implantation (ACI)^3^ and variations thereof.^4^ Unfortunately, microfracture generates fibrous cartilaginous scar tissue and therefore provides nonanatomic restoration of articular surface. ACI, and related techniques, have demonstrated superior mid- and long-term outcomes as compared with the simpler microfracture processes.^5^ However, culturing of chondrocytes in a 2-dimensional environment to obtain sufficient cells for implantation can lead to changes in the chondrocyte phenotype.^6,7^ Furthermore, substantial numbers of chondrocytes are harvested from an otherwise intact articular area creating additional damage in the joint surface.^6,8^

To reduce the number of chondrocytes (CHs) required for cell implantation, a combination of chondrocytes and mesenchymal stromal cells (MSCs) has been studied.^9^ Wu *et al*.^10^ demonstrated a beneficial effect on cartilage formation over the respective monocultures. MSCs increased chondrocyte proliferation and stimulated deposition of cartilage matrix. However, the trophic effect generated by MSCs is followed by a counter loop where the chondrocytes signal the MSCs to undergo cell death. This mechanism has been confirmed using a variety of MSC sources both *in vitro* and *in vivo* and in a clinical trial in which cartilage defects were implanted with a mixture of preoperatively isolated chondrocytes and allogenic bone marrow derived–stem cells.^11-14^

It was postulated that the mechanism behind the death of the MSCs is likely related to one of the deliberate “suicide” programs present within cells.^15^ These suicide programs are usually induced intrinsically or extrinsically by external stimuli such as mechanical stress, oxidative processes, and drug treatments. The programmed suicide death has usually 2 main forms, apoptosis and autophagy.^16-18^ These 2 pathways are intricately interconnected and the upregulation of one leads to downregulation of the other.^19^

Apoptosis is triggered by biochemical events that induce characteristic changes in the morphology of the cell (i.e., membrane blebbing and nuclear fragmentation). The apoptotic process can be intrinsically activated by the release of cytochrome *c* from mitochondria or can be activated extrinsically by death receptors. Both pathways lead to the activation of a series of caspases which mediate the cell destruction.^16^

Autophagy, is a catabolic process where the cell degrades cytoplasmic components important for survival, thereby leading to a so called “self-eating” phenomenon. This organized degradation and recycle activity uses vesicles, known as autophagosomes, which can contain organelles, proteins, and other components. These autophagosomes subsequently fuse with lysosomes that degrade both the cargo and the vesicles.^19^

In light of these results, we decided to investigate which of the 2 molecular mechanisms was involved in the possible cell death of MSCs in cocultures. Pellets containing the combination of human chondrocytes and human MSCs were cultured for 1 week and analyzed for changes in gene and protein expression characteristic for the apoptotic or autophagy pathways. Our data show a clear prevalence in activation of the autophagic pathway. We hypothesize that this mechanism could be a self-sacrifice mechanism of the MSCs which could contribute to the trophic effect of these cells on chondrocytes.

## Materials and Methods

### Cell Culture and Expansion

Human adipose tissue–derived MSCs were extracted from lipoaspirates obtained from consenting 2 male healthy donors (A211 and A283) and 1 female donor (A258) (Supplementary Table 1) as previously described.^20-22^ Chondrocytes were extracted from healthy looking cartilage of a donor undergoing an amputation. The use of cells for this study were approved by the Mayo Clinic Institutional Review Board.^22^

MSCs were cultured in standard medium (Gilco’s advanced modified Eagle medium; MEM) supplemented with 1% penicillin-streptomycin, 1% GLUTAmax, 5% human platelet lysate PLT max, and 0.2% heparin. Chondrocytes were cultured in chondrocytes proliferation medium (Gilco’s advance MEM, 10% fetal bovine serum [FBS], 1% penicillin-streptomycin, 1% GLUTAmax, 0.2 mM ascorbic acid 2-phosphate, and 4 mM proline). Both cell types were cultured in normal oxygen conditions (21% oxygen). MSCs were used at passage 5 and primary chondrocytes at passage 4.

### Pellet Coculture

Cell pellets were generated by seeding 200,000 cells per well in a 96-well plate. Cells were cultured as monocultures or as cocultures with a ratio of 80% MSCs/20% chondrocytes. Both mono- and cocultures were cultured in chondrogenic proliferation media (same as above) under hypoxia (2% oxygen). Medium was changed every three days. Pellets were harvested at day 7 for RNA-seq and immunofluorescence analysis.

### RNA Extraction

Total RNA was isolated from the samples using the Direct zol RNA kit as instructed by the manufacturer (Zymo Research, Irvine, CA). For each condition, 4 to 5 cell pellets were pooled to obtain sufficient RNA yield for downstream analysis. Nanodrop (Thermo Fisher Scientific, Waltham, MA) was used to determine the purity and concentration of the RNA extracted.

### RNA Sequencing and Analysis

RNA sequencing and bioinformatic analysis was performed by the Mayo Clinic RNA sequencing and bioinformatic cores as described previously.^22-25^ RNA libraries were prepared using the TruSeq RNA library preparation kit (Illumina, San Diego, CA) following the manufacturer’s instructions. The poly-A mRNA of each sample was purified from the total RNA using oligo dT magnetic beads. To multiplex sample loading on the flow cells, specific indexes were incorporated at the adaptor ligand using the TruSeq kit. The constructs were purified and enriched using 12 cycles of PCR. Agilent Bioanalyzer DNA 1000 chip and Qubit fluorometry (Invitrogen, Carlsbad, CA) were used to control the quality and the concentration of the samples. Libraries were loaded onto flow cells at concentrations of 8 to 10 pM to generate cluster densities of 700,000/mm^2^ following the standard protocol for the Illumina cBot and cBot Paired end cluster-kit version 3. Flow cells were sequenced as 51 X 2 paired end reads on an Illumina HiSeq 2000 using TruSeq SBS sequencing kit version 3 and HCS v2.0.12 data collection software. Base-calling was performed using Illumina’s RTA version 1.17.21.3. The RNA-Seq data were analyzed using the standard RNA-Seq workflow by Mayo Bioinformatics Core called MAPRSeq v.1.2.1, which includes alignment with TopHat 2.0.6^26^ and quantification of gene expression using the HTSeq software.^27^ Normalized gene counts were also obtained from MAPRSeq where expression values for each gene were normalized to 1 million reads and corrected for gene length (Reads Per Kilobase pair per Million mapped reads, RPKM). RNA-seq data were deposited in the Gene Expression Omnibus of the National Center for Biotechnology Information (GSE142831).

### Identification of Coculture Regulated Genes

To estimate the relative RNA contribution of the MSCs in the cocultures, we used the sex mismatch between the male donors (A211 and A283) and the female chondrocyte donor. The average level of expression of 7 unique male markers in the coculture was 57% of the levels in the respective monocultures (**Table 1**). As expected, the Y-markers were not expressed in the female chondrocytes and MSC (A258). We used these numbers to calculate the expected value of gene expression of a given gene using the following formula for each donor pair and calculated the average of the 3 donors. Only genes with RPKM values above 0.3 in each of the samples were included in the analysis.

(1) $$\begin{array}{l} RPKM\;Coculture_{expected}=0.57*RPKM\;AMSC_{mono}\\ \;\;\;\;\;\;+0.43*RPKM\;hCh_{mono} \end{array}$$

The expected value is valid under the assumption that gene expression in cocultures is the sum of gene expression in MSCs and chondrocytes and is not influenced by the interaction between both cell types.

**Table 1.** RPKM Values of 7 Individual Male Genes.^a^

Chr	GeneID	A211	A211 + ch	A283	A283 + ch	ch		([A211 + ch]/A211) * 100	([A283 + ch]/A283) * 100
chrY	RPS4Y1	108.6745	67.97581	92.28269	43.43297	0.337399		62.54994	47.06513
chrY	DDX3Y	21.43654	11.931	15.98953	8.763549	0.077968		55.6573	54.80805
chrY	PRKY	6.416036	3.24354	4.049743	2.343155	0.056736		50.55364	57.85934
chrY	USP9Y	3.647092	1.915803	1.484446	0.86582	0.023051		52.5296	58.32615
chrY	KDM5D	3.099356	2.289926	2.886254	1.85711	0.00957		73.88392	64.34326
chrY	ZFY	3.287543	1.731643	3.220577	1.739003	0.02219		52.67286	53.99662
chrY	EIF1AY	8.868548	4.7055	8.95836	5.544899	0.038423		53.05829	61.89636
							Average	57.27222	56.89927

RPKM = Reads Per Kilobase pair per Million mapped reads.

a The values of the monoculture are compared with the coculture and an average of the 7 genes was obtained. Based on these data, we assumed that 57% of the RPKM in the cocultures was derived from the mesenchymal stromal cells and 43% was derived from chondrocytes.

The real value was then compared with the expected value to determine the fold change (Fc) difference.

(2) $$Fc=\frac{RPKM\;Coculture_{real}}{Coculture_{expected}}$$

The majority of genes have an Fc of around 1 indicating that the observed gene expression is the sum of the expression in the MSCs and chondrocytes. Genes with an Fc > 2 cutoff were considered upregulated genes. Genes with an Fc < 0.5 were considered downregulated genes.

### Immunofluorescence Staining

Pellets were harvested for immunofluorescent staining as previously described.^28^ Cell-pellets were washed with phosphate-buffered saline (PBS) and fixed with 10% formalin for 15 minutes. Samples were then embedded in cryomatrix (Thermo Fisher) and cut into 10-μm sections with a cryotome (Shandon). Sections were permeabilized with 0.5% Triton X-100 in PBS for 10 minutes at room temperature followed by animal serum treatment (5%, 1 hour, room temperature) to block nonspecific binding. Sections were incubated overnight at 4°C in a humidified chamber with antibodies against LC3B (1:500 dilution, MAB85582, R&D System) and SQSTM1/p62 (1:200 dilution, ab56416, Abcam). Subsequently, slides were washed with 0.1% Tween 20 in PBS and incubated with Alexa Fluor-conjugated secondary antibodies (Alexa 568 or Alexa 488, Abcam) for 50 minutes at room temperature in a humidified chamber. Nuclei were counterstained with 4,6-diamidino-2-phenylindole (DAPI, Molecular Probes) and images were taken with a fluorescence microscope (Nikon Eclipse E400).

## Results

### Transcriptome Changes in MSCs and Chondrocyte Cocultures

The relative contribution of the MSCs in gene expression in the cocultures dropped from 80% (based on seeding ratio) to 57% after 1 week in coculture (**Table 1**). This confirms the disappearance of MSCs from cocultures and is in agreement with previous observations.^9^

**Figure 1.**
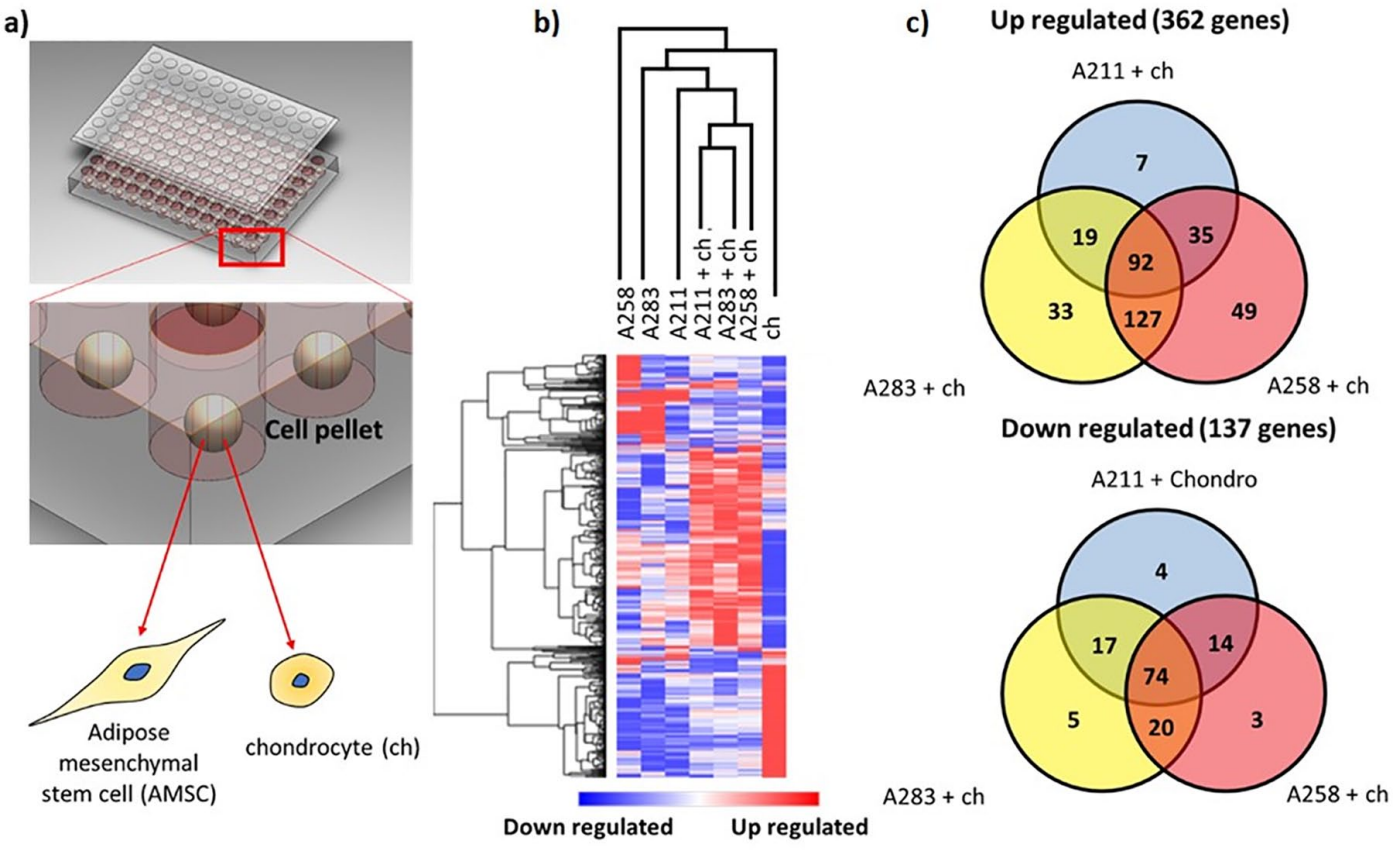
(**a**) A 96-well plate containing the monoculture or coculture pellets. (**b**) Heatmap of the entire genome normalized and clustered using MORPEUS software. The downregulated genes are expressed in blue whereas the upregulated in red. (c) Venn diagrams presenting the similarity between the upregulated (top) or down-regulated (bottom) genes belonging to the different cocultures.

Cluster analysis of RNAseq data from monocultured MSCs, chondrocytes, and cocultures at day 7 shows clustering of the distinct conditions in their respective groups (**Fig. 1b**). There is visible variation present in heatmap patterns when comparing the 3 MSC donors, indicative of interdonor variation.^29^ Moreover, the up- and downregulated genes in the hCHs and MSCs in the monoculture differ greatly from the coculture suggesting an interaction between the 2 cells. Based on the similarity in gene expression patterns between the 3 cocultures, it is likely that common pathways are influenced by the interaction between the MSCs and the chondrocytes.

A total of 362 genes met the inclusion criteria of >2-fold upregulation and *P* < 0.05 in at least one of the cocultures compared with the expected value assuming no cellular interaction between the MSCs and chondrocytes. In total, 137 genes were downregulated. The number of upregulated genes was nearly 3 times higher compared with the downregulated genes, which may indicate a predominance of pathway activation when cells are placed in coculture. A Venn diagram was created to identify common up- or downregulated genes in each of the cocultures. Cocultures of chondrocytes with donors A258 and A283 represented a closer pattern of gene up- and downregulation as compared with the coculture involving donor A211, which further highlights the presence of interdonor variability (**Fig. 1c**). In total, 92 genes were more than 2-fold upregulated in all 3 cocultures and 74 were more than 2-fold downregulated (Supplementary Figure 1).

### Upregulation of Autophagic and Apoptotic Pathways in MSCs and Chondrocyte Cocultures

**Figure 2.**
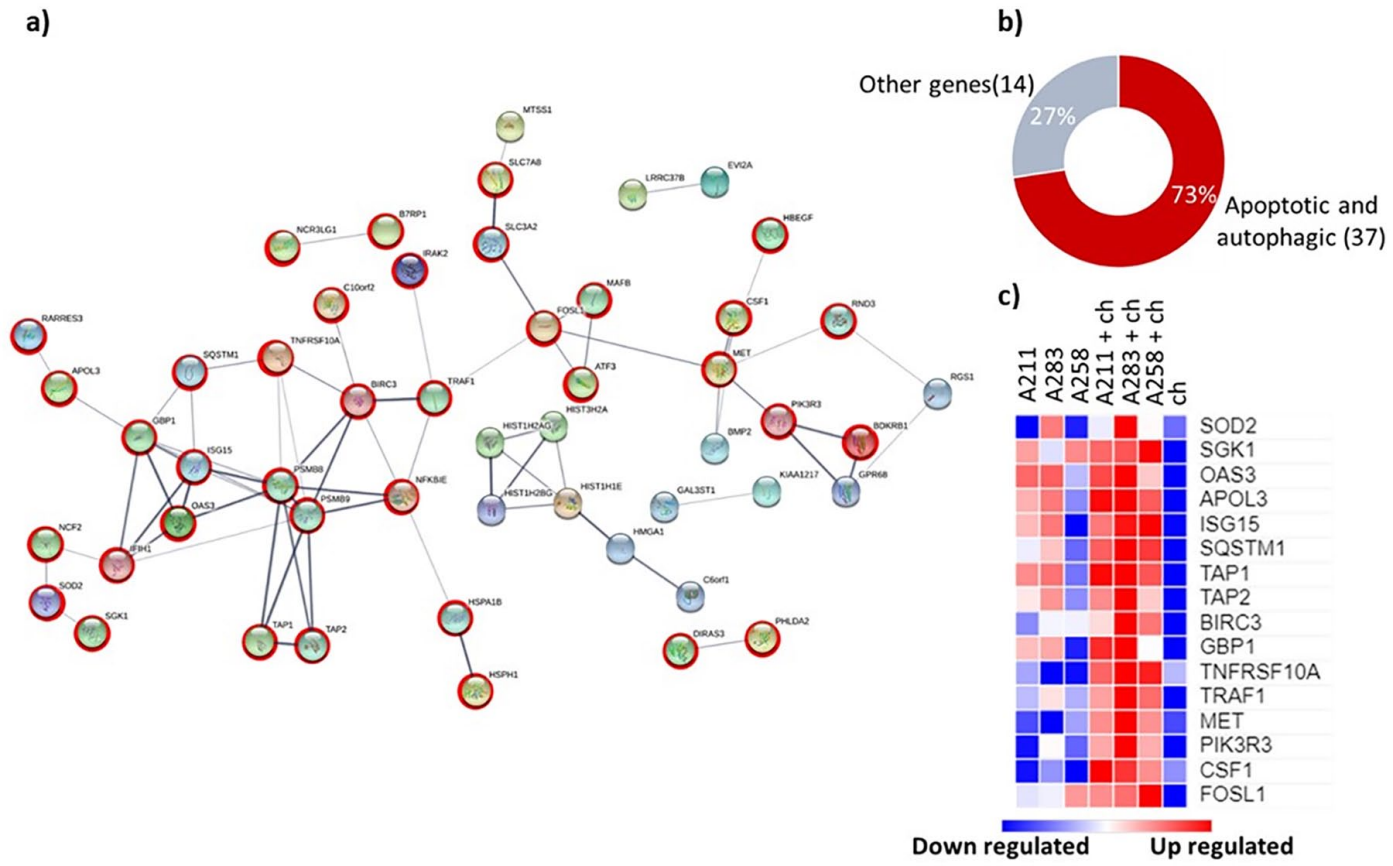
(**a**) Interconnections of the 51 upregulated genes using STRING software. Red circle symbolizes genes that have previously been annotated to apoptotic or autophagic processes. For other genes insufficient data were present in literature or have been previously annotated to other pathways. (**b**) Pie chart presenting the percentage of the 51 genes involved in the apoptotic or autophagic pathways based on gene counts in **a**. (c) Heatmap presenting the normalized (log 2) overexpression (red) levels of 17 upregulated genes using the RPKM values obtained from the RNA sequencing.

Among the 92 upregulated genes, 86 were identified (Supplementary Table 6) by the STRING software and 51 were found having known interactions (medium confidence 0.4). Two main clusters were obtained: one consisting of a series of histones (HIST1H2AG, HIST1H2BG, HIST3H2A, HIST1H1E) which may indicate an effect on cell proliferation in line with previous observations.^10^ The second, a larger cluster of 51, consisted of a series of genes mainly present in cell death processes like autophagy and apoptosis (37 out of 51 genes^16,30^) (**Fig. 2**). The genes identified encode surface (RARRES3,^31^ TNFRSF10A,^32^ PIK3R3,^33^ TRAF1,^34^), cytoplasmic (NCF2,^35^ APOL3^36^) and transporter (TAP1, TAP2)^37^ proteins. A literature search of the 51 genes confirmed their role in cell death pathways in more detail (Supplementary Table 2). In general, most of the GO-terms obtained from the ClueGO analysis represented terms like cell stress or death pathways (**Fig. 2** and Supplementary Table 3). Moreover, 52.17% of the terms GO-pathways detected were related to ubiquitin-specific processing proteases. Ubiquitination represents a fundamental process in the autophagic machinery.^38^ These data suggest that the coculturing of MSCs and chondrocytes may induce autophagic cell death.

### Coculture Treatment Induces Autophagic Flux

**Figure 3.**
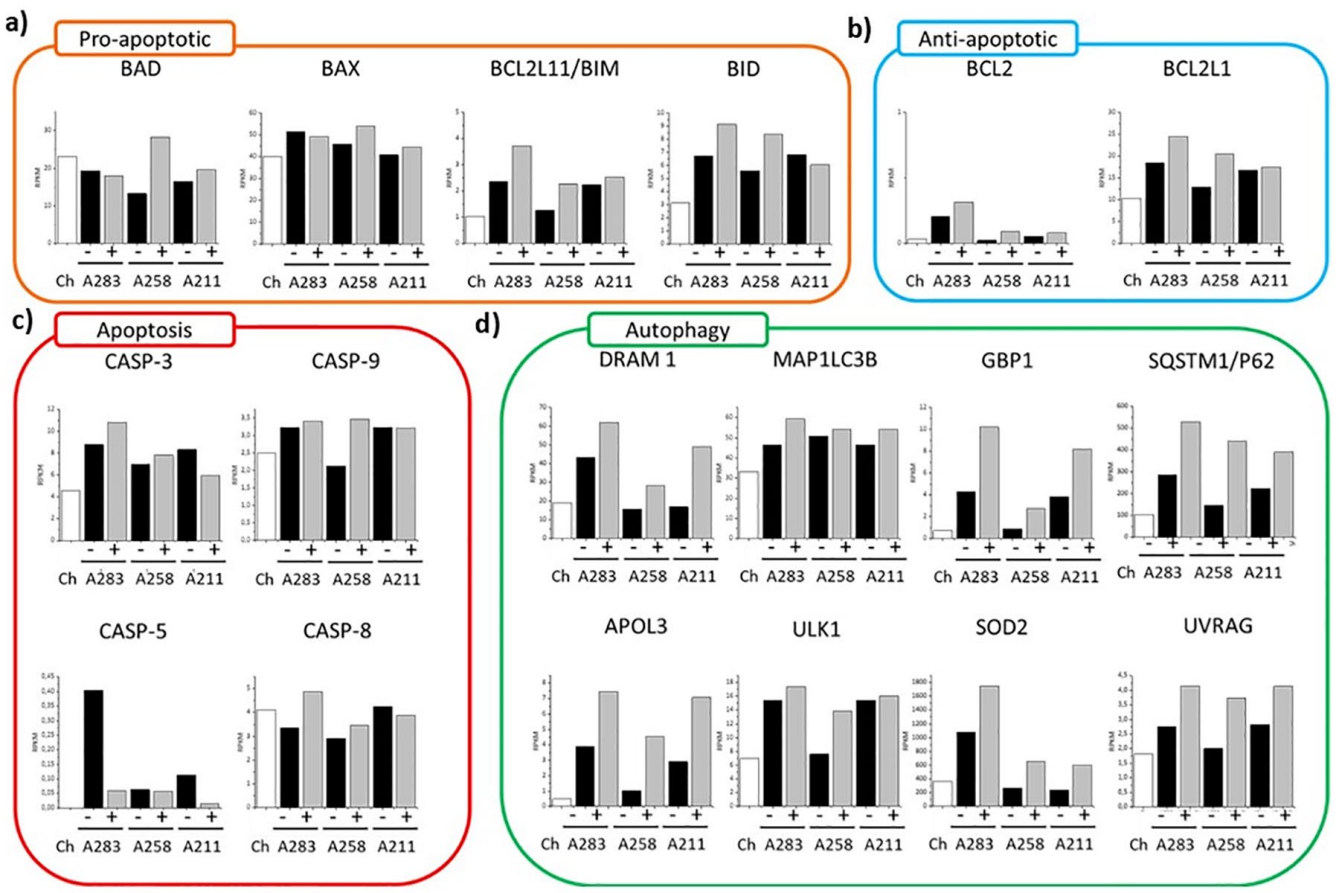
(**a**) Comparison between the RPKM values of pro-apoptotic markers (BAD, BAX, BCL2L11/BIM, BID); (**b**) of the anti-apoptotic markers (BCL2, BCL2L1); (**c**) apoptotic markers (CASP-3, CASP-9, CASP-5, CASP-8); (**d**) of the autophagic markers (DRAM1, MAP1LC3B, GBP1, SQSTM1/P62, APOL3,ULK1, SOD2, UVRAG) obtained from the RNA sequencing.

To further distinguish whether the MSCs disappear from coculture by apoptosis or autophagy, the expression of pro-apoptotic, anti-apoptotic, apoptotic, and autophagic markers was assessed (**Fig. 3a-d**).

**Figure 4.**
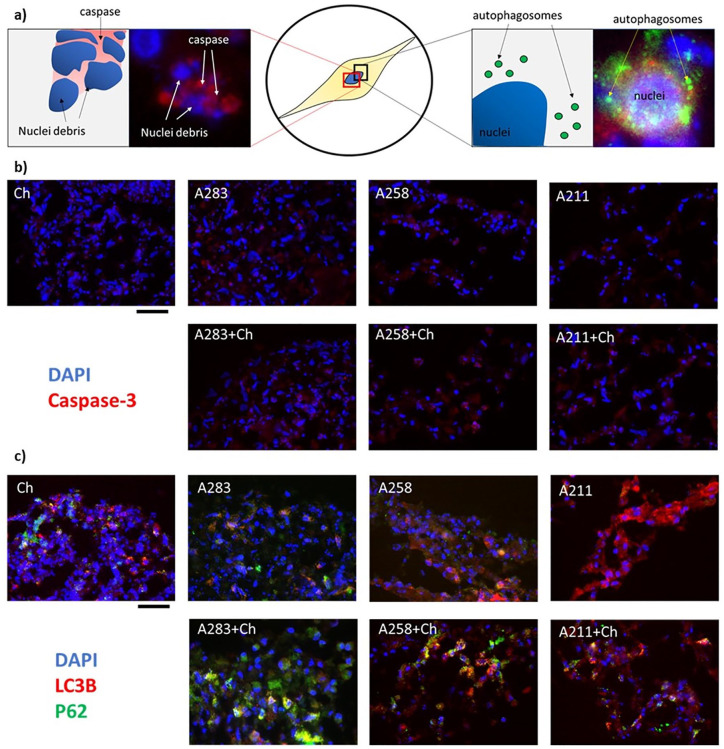
(**a**) Magnification of a cell undergoing apoptosis (on the left) through caspase-3 (in red) or autophagy (on the right) through LC3B (red) and P62 (green). In blue nuclei (**b**) immunofluorescence of the 7 conditions after 7 days of culture of the caspase-3 (in red) and nuclei (in blue); (**c**) immunofluorescence of the same 7 conditions of LC3B (in red) and P62 (in green). In blue nuclei. Yellow color represents overimposed green and red signals. Scale bars equate to 40 μm.

First, we looked at 4 pro-apoptotic markers (BAD, BAX, BIM, BID) (**Fig. 3a**). These markers belong to the BCL-2 cell-death-regulator-family and they initiate and/or mediate the activation of apoptosis. Here, by comparing the monoculture with the coculture, the variability among the MSCs donors is clear. Some of the genes are overexpressed in mono- or coculture depending on the donor. However, the trends between the 2 conditions do not present any statistical difference, which suggests inactivation of the apoptotic pathway (Supplementary Table 4). This is furthermore supported by the gene expression of four of the main caspase pathway regulators (*CASP3*, *CASP5*, *CASP8*, *CASP9*)^16^ that did not change or were less expressed in cocultures (**Fig. 3c**). Moreover, immunofluorescence images were obtained to determine the level of caspase-3 (**Fig. 4b**). Caspase-3 is the final protein of the apoptotic cascade cycle.^39^ During apoptosis, the cells present disrupted nuclei comprising high level of caspase-3 (**Fig. 4a**, left). However, in both mono- and coculture, this characteristic is barely present indicating absence of apoptotic activation.

We further checked 2 of the main anti-apoptotic markers (BCL2 and BCL2L1). Although both did not reach a statistical significance (0.11 BCL2 and 0.15 BCL2L1), both presented, for each of the 3 donors, an increase in expression in coculture (**Fig. 3b** and Supplementary Table 4). This suggests an activation of anti-apoptotic processes, which may cause the in-activation of the caspase cycle.

Furthermore, as regulated necrosis can be triggered by binding of TNF-α and FAS ligand we looked at overexpression of RIPK1, RIPK3, and MLKL. However, none of these genes were significantly upregulated in the cocultured compared with the monoculture (Supplementary Fig. 4) excluding a role for necrosis in the disappearance of the MSCs.

We next determined the effects of autophagic markers in the cocultures (**Fig. 4**). First, we looked at multiple markers highly present during autophagy activation (**Fig. 3d**). Among the 8 individual markers, 7 were statistically upregulated in the coculture compared with the monoculture, indicating activation of the autophagic machinery at the gene level. We further looked at the protein level using immunofluorescence. Here, 2 well-recognized markers (LC3B and P62) were used. LC3B is conjugated to the autophagosome during autophagosome formation. P62/SQSTM1 protein interacts with both LC3B-II and ubiquitin protein and is degraded in autophagolysosomes.^40^ In in-activated autophagy, these 2 markers can be singularly present (Supplementary Fig. 3 A211) or can be present together but not colocalized (Supplementary Fig. 3 A258). On the contrary, in active autophagy both markers are present and colocalize (**Fig. 4a**), which indicates creation of the autophagosome. Even if the behavior of the 3 donors is different, it is clear that the level of combined LC3B and P62 are higher in the coculture compared with the monoculture for all the donors, indicating upregulation of the autophagic machinery.^41^ Taken together, these results suggest that MSCs exhibit enhanced autophagic flux.

## Discussion

In this study, we have studied the mechanism involved in the progressive cell death of the MSCs in coculture with chondrocytes.

We used the sex mismatch between the female chondrocyte and male MSC donors to estimate the relative contribution of the MSCs to the gene expression in the cocultures. The expression of Y-chromosome-specific RNAs proved stable across donors. By assuming that the expression of Y-specific markers is not influenced by the coculture conditions, a notion which is supported by previous observations, it is possible to estimate the contribution of the male MSC donors to global RPKM in coculture with female chondrocytes. Using this approach, we concluded that the relative contribution of MSCs to the RPKM in the cocultures dropped from 80% to 57% after 1 week of culture. This supports previous observations where after 4 weeks of culture, MSCs have almost completely disappeared from cocultures with chondrocytes due to cell death irrespective of the origin of the stem cells.^9^ Signs of increased cell death were first noted at day 7 and increased at day 14.^10^ We reasoned that the 7-day time point marked the beginning of the disappearance of MSCs from cocultures and selected this time point for an RNA-seq analysis. At this time point, the transcriptome of the cocultures was substantially and statistically different from the respective monocultures indicating the presence of nonadditive interactions between the 2 cell populations. 362 genes were upregulated of 2-fold in at least 1 of the cocultures. Of these genes, 92 were consistently upregulated in each of the 3 cocultures. The considerable interdonor variability is in line with previous studies.^42^ Interestingly, the donor A258 (female) and A283 (male), presents higher overlap in terms of both up- and downregulated genes in coculture suggesting that the trophic role of MSCs is a generic, sex-independent property of these cells as noted before.^43^ We do realize that the method for selecting genes specifically regulated by the interaction between chondrocytes and MSCs has limitations. For example, genes that are inversely regulated in MSCs and chondrocytes may be missed. The genes identified in this study thus represent a snapshot of genes that are regulated in cocultures.

STRING analysis identified 2 interconnected networks of which the main included more than half of the total upregulated genes. This network describes genes involved in apoptotic or autophagic processes and comprised proteins involved in subsequent steps of these pathways starting from membrane receptor proteins (TNFRSF10A, PIK3R3, TRAF1) up to fundamental enzymatic components (OAS3). Most of the genes detected are upregulated in one (RARRES3), or both processes (DIRAS3, ISG15, BMP2, PHLDA2). Other genes are involved in the inhibition of one (HBEGF-for apoptosis) or both pathways (ATF3). Moreover, genes such as BIRC3 and NCF2 can upregulate one process while inhibiting the other. Interestingly, these results differ from those of Wu *et al*.^44^ where a microarray analysis identified clusters related to intracellular cell cycle regulators, extracellular matrix production and secreted growth factors (FGF1 and BMP2). This variability could be related to the time points considered. Wu *et al*.^44^ performed the analysis at the second day of culture, whereas our study focused on a later time point (day 7). At this earlier time point, a dominant effect on cell proliferation was found whereas the disappearance of MSCs from the cocultures was first noted at day 7.^9,10^ Also, in our study, we show the upregulation of many proliferation markers, however, this upregulation was modest and consequently did not meet the cutoff of 2-fold used (Supplementary Table 7). No upregulation of cartilage matrix genes was noted, which could be explained by the early time point considered. Interestingly, a decrease in COL10A1 and COL3A1 mRNA expression was visible indicating a reduction in the hypertrophic activity (Supplementary Fig. 5).

Like in the study of Wu *et al*.,^44^ we have found upregulation of BMP2. This suggests that BMP2 plays a fundamental and prolonged role in the coculture effect. This contrasts FGF1, which in this study was not found upregulated. Combined this data may suggest that FGF-1 functions as trigger in an initial phase in chondrocyte proliferation, whereas BMP-2 is required for longer period of time and may sustain cartilage matrix formation.^45-48^ Alternatively, the differences could be explained by the use of adipose MSCs versus bone marrow MSCs in the study by Wu *et al*.^44^ This seems, however, unlikely given the consistency in the trophic effect of MSCs from a variety of sources in coculture with primary chondrocytes.^9^ Moreover, the use of a more physiologically relevant hypoxic environment, used in this study, compared with the normoxic environment in the study by Wu *et al*.,^44^ may also have contributed to the differences in gene expression.

Apoptosis can be initiated by an extrinsic or, an intrinsic process both ending with the cleavage of the procaspase-3.^16^ Here, the RNA expression data suggested the possible activation of the extrinsic pathway by membrane receptor proteins (TRAF1, TNFRSF10A) rather than the activation of the intrinsic pathway by genes like DIABLO, HTRA2, AIFM1, ENDOG, and CAD (Supplementary Table 5). Since we did not found an in increase or difference in the active form (caspase-3 within the nuclei and nuclei debris) at the protein level, we concluded that the cell death via the canonical apoptotic pathway is likely not driving MSCs death.

Autophagy is a cellular degradation pathway that is essential for survival.^49^ However, if overexpressed, it could lead to neurodegeneration, cardiomyopathies, abnormalities of skeletal development, and death as shown in mice studies.^18,50^ LC3-II is as a quantitative marker of autophagy required for the formation of the autophagosome and its expression is proportional to the amount of autophagosomes in the cell. The P62/SQSTM1 protein serves as a link between LC3 and ubiquitinated substrates.^51^ P62/SQSTM1 and P62-bound polyubiquitinated proteins become incorporated into the completed autophagosome. From the data obtained, LC3-II and P62/SQSTM1 were highly expressed, both at the gene and protein level, in the cocultures compared to the monoculture conditions suggesting activation of the autophagic flux. Taken together, our data shows a strong association between the activation of autophagy and the disappearance of MSCs from cocultures with chondrocytes suggesting that the MSCs in coculture preferentially die by autophagy rather than by apoptosis. Formal proof of this hypothesis would require further studies for example by knocking down genes involved in the autophagic and apoptotic pathways.

This conclusion differs from the previous study performed by Wu *et al*.^10^ where high levels of TUNEL positivity staining were detected in the pellets. TUNEL staining detects the DNA breaks formed when DNA fragmentation occurs in the last phase of apoptosis. Normally, this kind of apoptosis is considered as caspase-dependent procedure. However, cell death can proceed in caspase-independent apoptotic pathway, in which TUNEL positive staining is also observed.^52^ The mitochondria play a central role in both caspase-dependent and caspase-independent death pathways.^53^ It has been recognized that mitochondria can release factors involved in caspase-independent cell death, including apoptosis-inducing factor (AIF) and endonuclease G (EndoG).^54-56^ In fact, AIF is believed as a key mediator of poly ADP-ribose (PAR) polymerase (PARP) induced caspase-independent cell death.^57^ Indeed, it has been reported that autophagy is a cytosolic event that controls caspase-independent macrophage cell death through RARP mediated pathway.^58^ Moreover, autophagy activated by DNA damage can kill the cells through the autophagy regulators in the absence of apoptosis.^59-61^ However, the major concern with these examples is that they represent a very artificial situation. Regardless, these data may explain the difference between these 2 studies.

It remains unclear which role MSCs cell death by autophagy plays in the coculture. We hypothesize that the autophagic extracellular vesicles generated may have an additional trophic effect. During autophagy, cells release a variety of signals, including extracellular vesicles, which after uptake by neighboring cells, induce cellular responses over short- and/or long-range distances.^62^ Indeed, researchers proposed the concept of “altruistic cell suicide” based on the observation that dying cells could induce proliferation of neighboring cells.^63^ Based on our analysis, it is conceivable that the activation of autophagy in MSCs likely initiates this “altruistic cell death” process in coculture with chondrocytes. We propose a sequential mechanism where growth factors (FGF1 and BMP2) are released by the MSCs and subsequently are further enhanced by the increased secretion of extracellular vesicles, and their uptake by neighboring chondrocytes. This mechanism can explain how MSCs stimulate cartilage formation while simultaneously disappearing both *in vitro* and *in vivo*.

Additional studies should focus on the mechanism behind the initiation of autophagy and genetic interference studies using, for example, knock down approaches, the release of extracellular vesicles, and their uptake. Particularly interesting are the studies aimed at analyzing the content of the autophagic vesicles. Activation of autophagy in MSCs might be an efficient way to increase the formation of trophic extracellular vesicles. This may help in optimizing intra-articular injection strategies based on MSC-derived extracellular vesicles rather than MSCs themselves.^64-66^ The use of MSC-derived extracellular vesicles rather than the cells themselves may avoid possible long-term phenotype changes of incorporated cells and attenuate many of the safety concerns related to the use of living cells.

## Conclusion

In summary, here we provide evidence that MSCs in coculture with primary chondrocytes preferentially die by autophagy. We postulate that this altruistic cell death results in the formation of extracellular vesicles. These extracellular vesicles are an additional mechanism by which the MSCs stimulate chondrocyte proliferation and cartilage matrix formation in pellet cocultures.

## Supplemental Material

supinfofinalfinalversion_adapted_for_resub_complete – Supplemental material for Autophagy Is Involved in Mesenchymal Stem Cell Death in Coculture with Chondrocytes Click here for additional data file. Supplemental material, supinfofinalfinalversion_adapted_for_resub_complete for Autophagy Is Involved in Mesenchymal Stem Cell Death in Coculture with Chondrocytes by Carlo Alberto Paggi, Amel Dudakovic, Yao Fu, Catalina Galeano Garces, Mario Hevesi, Daniela Galeano Garces, Allan B. Dietz, Andre J. van Wijnen and Marcel Karperien in CARTILAGE
