# Supplementary material for: Autophagy Is Involved in Mesenchymal Stem Cell Death in Coculture with Chondrocytes
Source: Cartilage. 2020 Jul 22;13(2 Suppl):969S–979S. doi: 10.1177/1947603520941227 (PMC8721613; doi:10.1177/1947603520941227)
Supplement: supinfofinalfinalversion_adapted_for_resub_complete – Supplemental material for Autophagy Is Involved in Mesenchymal Stem Cell Death in Coculture with Chondrocytes [file supinfofinalfinalversion_adapted_for_resub_complete.pdf]

Supplementary information

Up-regulated genes

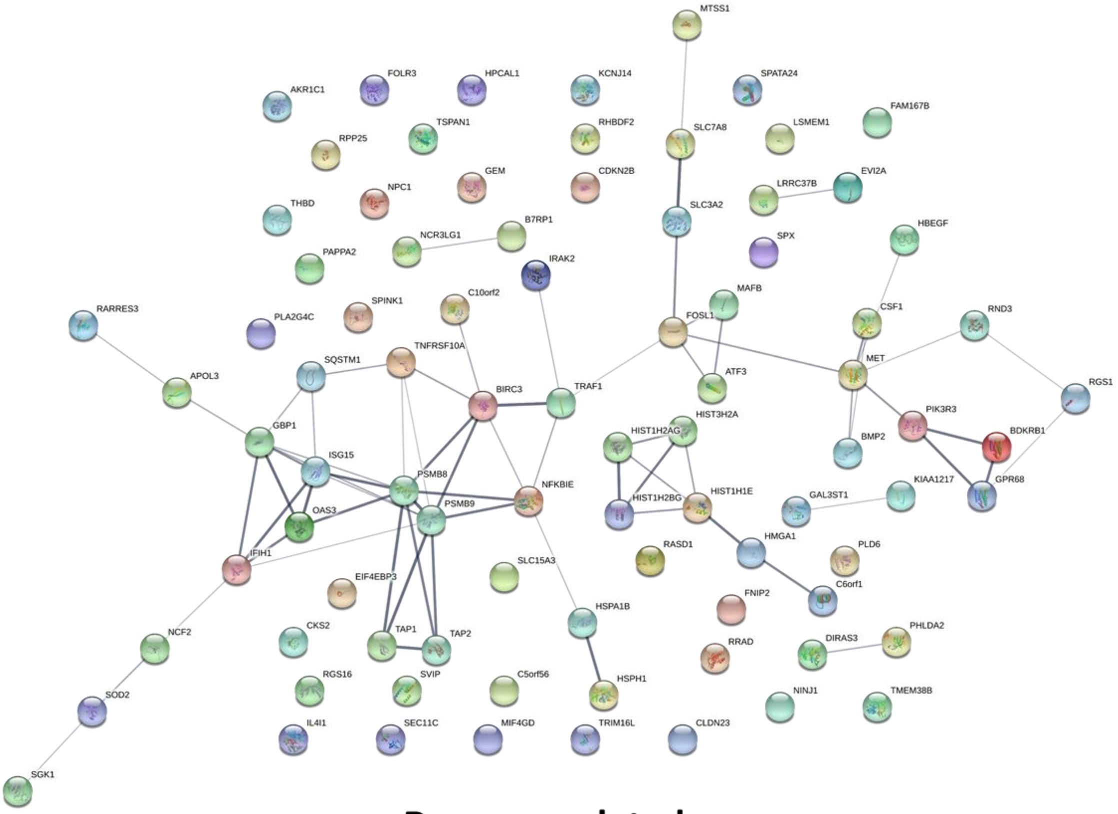

Down-regulated genes

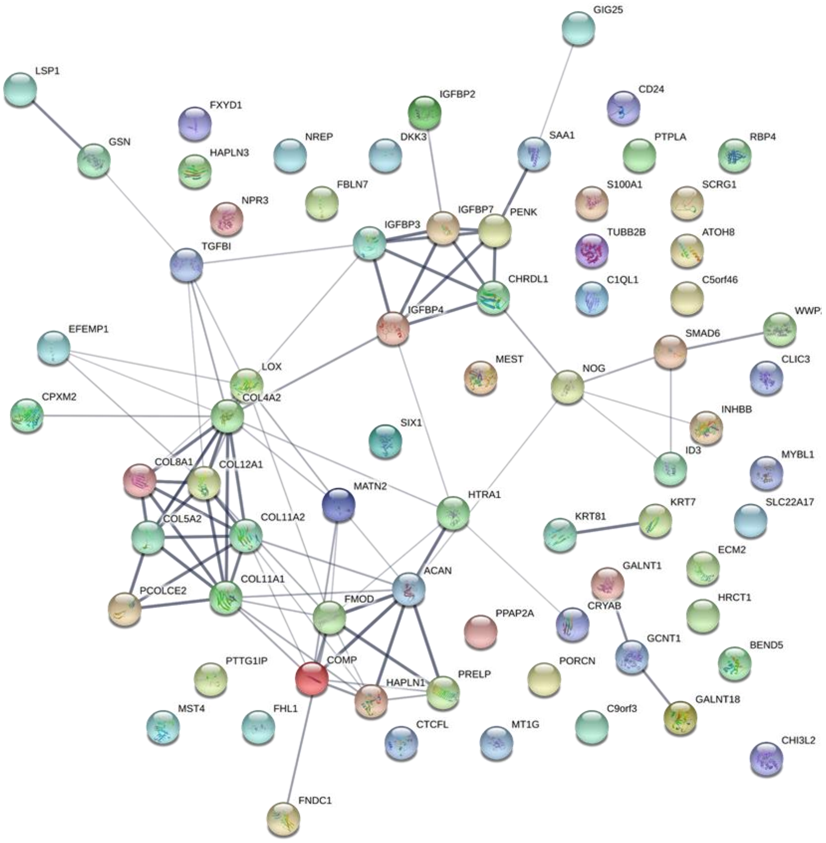

Supplemental Figure 1: Up- and Down-regulated genes using STRING software.

Supplemental Table 1: Scheme of donor variability in terms of sex, age, extraction position and level of diabetes.

AMSCs:	211	258	283
**Sex**	Male	Female	Male
**Age**	41	32	54
**Position**	abdominal area	upper thigh	abdominal area
**Diabetes**	Normal	Normal	Normal

Supplemental Table 2: Up-regulated genes having connection detected using STRING software. For each gene a literature search was performed to determine if the gene was actively involved in the autophagic or apoptotic machinery. In green genes enhancing the pathways. In red the genes inhibiting the pathways. In black not fully defined genes which were shown having an effect in autophagy &/or apoptosis.

SYMBOL	FULL NAME	AUTOPHAGIC &/OR APOPTOTIC PATHWAY
**MTSS1**	MTSS I-BAR domain containing 1	--
**SLC7A8**	Solute Carrier Family 7 Member 8	Apoptosis [1]
**SLC3A2**	Solute Carrier Family 3 Member 2	Apoptosis [2]
		Apoptosis[3]
		Autophagy (indirect) [4]
		Autophagy (indirect) [5]
**MAFB**	MAF bZIP transcription factor B	Apoptosis [6]
**ATF3**	activating transcription factor 3	Autophagy & Apoptosis [7]
		Autophagy [8]
		Autophagy [9]
		Apoptosis [10]
		Apoptosis [11]
		Apoptosis [12]
		Apoptosis [13]
		Apoptosis [14]
**LRRC37B**	leucine rich repeat containing 37B	--
**EVI2A**	ecotropic viral integration site 2A	--
**HBEGF**	heparin binding EGF like growth factor	Apoptosis [15]
		Apoptosis [16]
		Apoptosis [17]
		Apoptosis [18]
**RND3**	Rho family GTPase 3	Autophagy [19]
		Apoptosis [20]
		Apoptosis [21]
		Apoptosis [22]
**RGS1**	regulator of G protein signaling 1	Apoptosis [23]
**GPR68**	G protein-coupled receptor 68	Apoptosis [24]
**BDKRB1**	bradykinin receptor B1	Apoptosis [25]
**KIAA1217**		Apoptosis [26]
**BMP2**	Bone morphogenic protein 2	Autophagy [27]
		Autophagy [28]
		Apoptosis [29]
		Apoptosis [30]
		Apoptosis [31]
		Apoptosis [32]
**GAL3ST1**	galactose-3-O-sulfotransferase 1	Apoptosis [33]
**PHLDA2**	pleckstrin homology like domain family A member 2	Apoptosis [34]
		Apoptosis [35]
		Apoptosis [36]
		Apoptosis [37]
		Autophagy [38]
**DIRAS3**	DIRAS family GTPase 3	Apoptosis & autophagy [39]
		Autophagy [40]
		Autophagy [41]

		Autophagy [42]
		Apoptosis [43]
**C6ORF1**	small integral membrane protein 29	--
**HMGA1**	high mobility group AT-hook 1	Autophagy [44]
		Apoptosis [45]
		Apoptosis [46]
**HIST1H1E**	histone cluster 1 H1 family member e	Apoptosis [47]
		Apoptosis [48]
**HIST3H2A**	histone cluster 3 H2A	--
**HIST1H2AG**	histone cluster 1 H2A family member g	--
**HIST1H2BG**	histone cluster 1 H2B family member g	--
**B7RP1**	inducible T cell costimulator ligand	Apoptosis [49]
**NCR3LG1**	natural killer cell cytotoxicity receptor 3 ligand 1	Apoptosis [50]
		Apoptosis [51]
**HSPH1**	heat shock protein family H (Hsp110) member 1	Apoptosis [52]
		Apoptosis [53]
		Apoptosis [54]
		Apoptosis [55]
		Apoptosis [56]
**HSPA1B**	heat shock protein family A (Hsp70) member 1B	Apoptosis [57]
		Apoptosis [58]
		Apoptosis [59]
		Apoptosis [60]
**RARRES3**	phospholipase A and acyltransferase 4	Apoptosis [61]
		Apoptosis [62]
		Apoptosis [63]
**IFIH1**	interferon induced with helicase C domain 1	Autophagy [64]
		Autophagy [65]
		Apoptosis [66]
		Apoptosis [67]
		Apoptosis [68]
**SGK1**	glucocorticoid-induced protein kinase 1	Apoptosis & autophagy [69]
		Apoptosis [70]
**SOD2**	manganese superoxide dismutase (Mn-SOD	Apoptosis & autophagy [71]
		Autophagy [72]
**NCF2**	Neutrophil Cytosolic Factor 2	Apoptosis & autophagy [73]
**TAP1**	Antigen peptide transporter 1	Autophagy [74]
		Apoptosis [75]
**TAP2**	Antigen peptide transporter 2	Autophagy [74]
**OAS3**	2'-5'-oligoadenylate synthetase 3	Autophagy {cannot find ref in endnote)
**ISG15**	Interferon-stimulated gene 15	Autophagy [76]
		Autophagy [77]
		Apoptosis [78]
**GBP1**	Guanylate-binding Protein 1	Apoptosis & autophagy [79]
**APOL3**	Apolipoprotein L3	Apoptosis & autophagy [80]
**SQSTM1**	Sequestosome 1	Autophagy [81]
		Apoptosis [82]
**PSMB8**	Proteasome Subunit Beta 8	Apoptosis & autophagy [83]

**PSMB9**	Proteasome Subunit Beta 9	Apoptosis & autophagy [83]
**BIRC3**	Baculoviral IAP Repeat Containing 3)	Apoptosis & autophagy [84]
**NFKBIE**	nuclear factor of kappa light polypeptide gene enhancer in B-cells inhibitor, epsilon	Autophagy [85]
**TRAF1**	TNF Receptor Associated Factor 1	Autophagy [86]
		Apoptosis [87]
**IRAK2**	interleukin 1 receptor associated kinase 2	Apoptosis [88]
**C10ORF2**	chromosome 10 open reading frame 2	Apoptosis [89]
		Apoptosis [90]
**FOSL1**	Fos-related antigen 1	Autophagy [91]
**CSF1**	colony stimulating factor 1	Autophagy [92]
		Apoptosis [93]
**MET**	MET Proto-Oncogene, Receptor Tyrosine Kinase	Autophagy [94]
		Apoptosis[95]
**PIK3R3**	phosphoinositide-3-kinase regulatory subunit 3	Apoptosis[96]
**TNFRSF10A**	Tumor necrosis factor receptor superfamily member 10A	Apoptosis & autophagy[3]

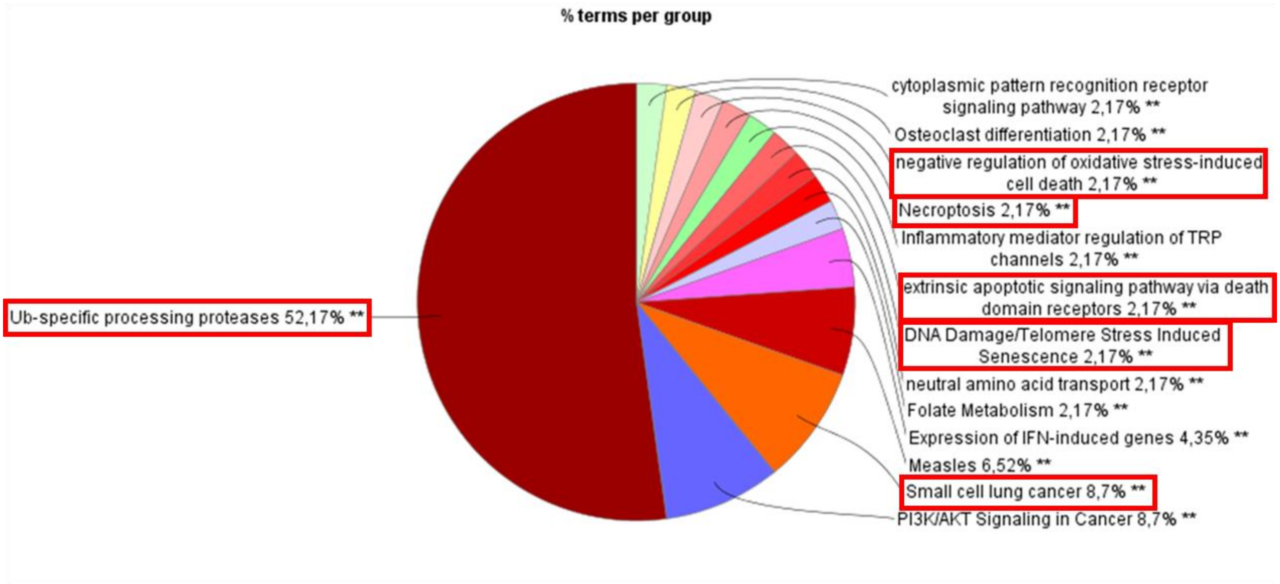

Supplemental Figure 2: Pie plot showing the % of terms per group of pathways detected. Each group is constituted by a number of GO-pathways (Table 3) discovered using ClueGO. The majority of the GO-pathways identified is involved one of the death machinery (red square). The same color is used for the group presented in this pie plot and for table 3.

Supplemental Table 3: GO-pathways of the 92 up-regulated genes detected using clueGO. The two small red stars above the bars indicate statistical significance (P-value<0.001).

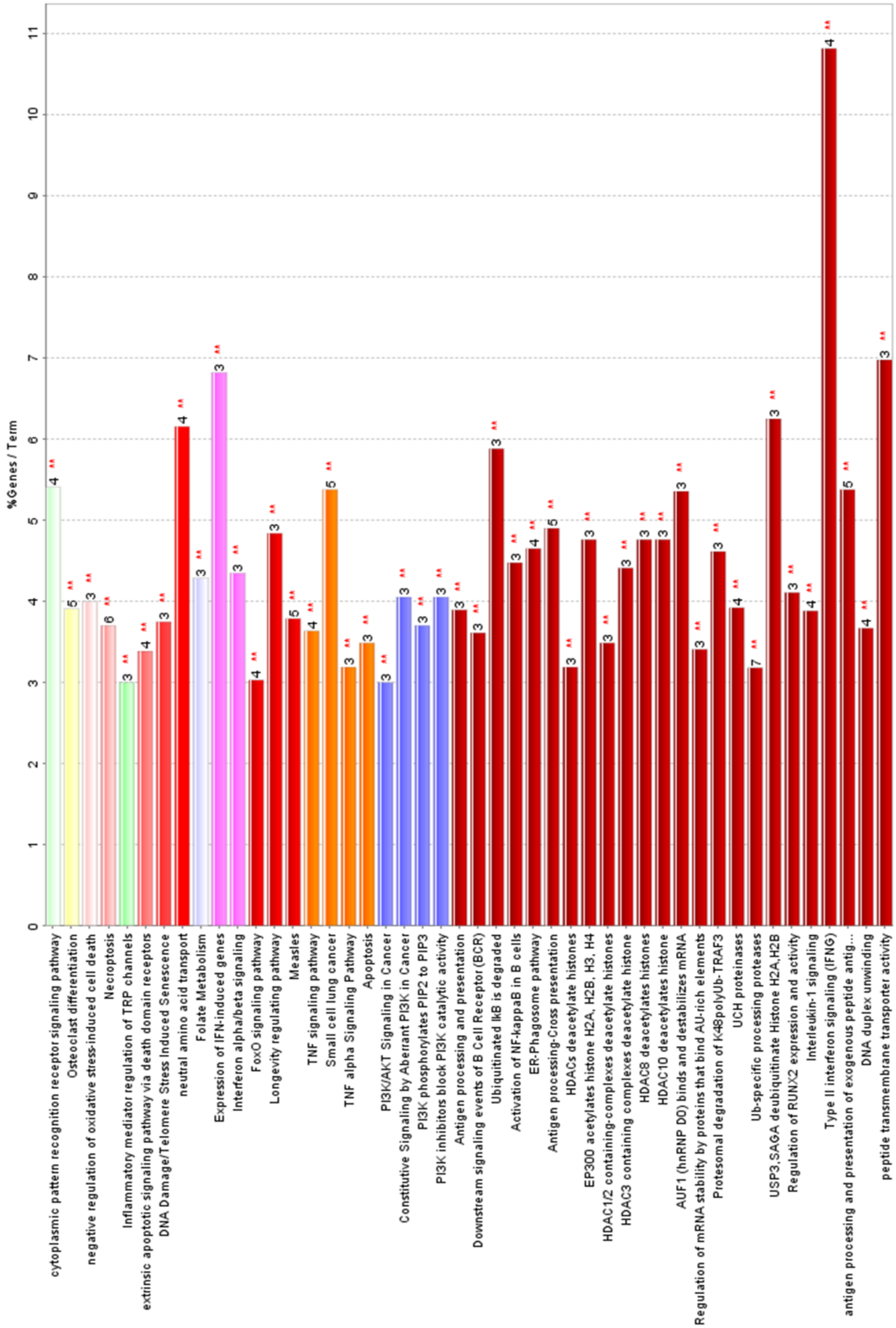

Supplemental Table 4: RPKM data of 18 individual genes related to autophagy, apoptosis, pro-apoptotic and anti-apoptotic machinery. The data of the MSCs mono-culture are then compared to the co-culture using a paired t-test to determine the statistical difference. In yellow, values statistically different (at least below 0.1).

GeneID	Comment	ch	A211	A283	A258	A211+ch	A283+ch	A258+ch	t-test (PAIRED)
DRAM1	Autophagy	19,19	17,00	43,39	15,94	49,08	62,15	28,27	0,069
SQSTM1/P62	Autophagy	105,40	224,13	284,98	146,92	390,61	529,99	441,07	0,024
MAP1LC3B	Autophagy	33,22	46,46	46,36	50,78	54,16	59,36	54,29	0,099
GBP1	Autophagy	0,75	3,84	4,31	0,88	8,19	10,22	2,76	0,075
APOL3	Autophagy	0,50	2,91	3,89	1,05	7,07	7,47	4,56	0,003
ULK1	Autophagy	6,95	15,43	15,39	7,59	16,07	17,38	13,83	0,222
SOD2	Autophagy	366,97	238,93	1074,04	268,41	603,86	1750,18	656,89	0,041
UVRAG	Autophagy	1,81	2,83	2,76	2,01	4,13	4,15	3,74	0,008
CASP3	Apoptosis	4,58	8,34	8,79	7,00	5,96	10,82	7,82	0,917
CASP5	Apoptosis	0,00	0,11	0,40	0,06	0,01	0,06	0,06	0,277
CASP8	Apoptosis	4,12	4,25	3,36	2,91	3,88	4,88	3,46	0,406
CASP9	Apoptosis	2,50	3,23	3,23	2,14	3,22	3,42	3,47	0,351
BAX	pro-apopotic	40,17	40,81	51,40	45,74	44,42	49,13	54,02	0,404
BAD	pro-apopotic	23,08	16,52	19,33	13,25	19,63	18,01	28,19	0,369
BCL2L11/BIM	pro-apopotic	1,04	2,25	2,37	1,26	2,53	3,72	2,28	0,109
BID	pro-apopotic	3,15	6,81	6,70	5,59	6,03	9,13	8,38	0,321
BCL2	anti-apoptotic	0,03	0,06	0,20	0,02	0,08	0,32	0,09	0,110
BCL2L1	anti-apoptotic	10,33	16,77	18,44	12,94	17,45	24,49	20,45	0,149

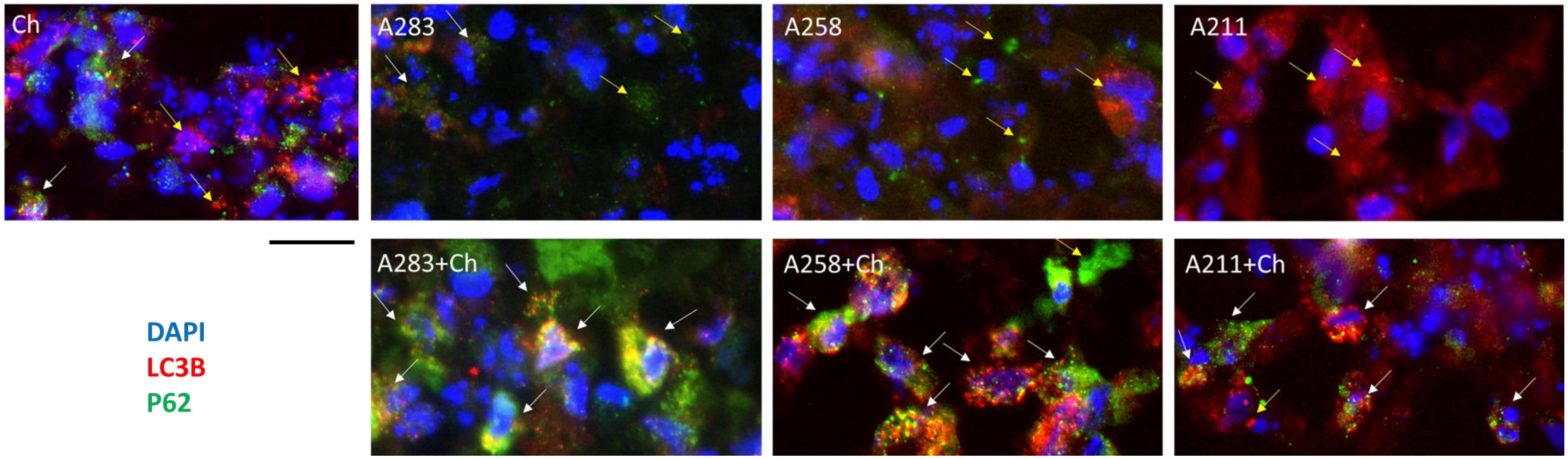

Supplemental Figure 3: Immunofluorescence of the 7 individual conditions. In blue DAPI, in red LC3B and in green P62. Yellow arrow indicates LC3B or P62 in an inactive state. White arrow indicates combination of LC3B and P62 and activation of the autophagic machinery. Scale bar 20 µm.

Supplemental Table 5: RPKM data of 7 individual genes initiating the apoptotic machinery. In light blue the genes initiating apoptosis through an intrinsic pathway in green two receptor genes of the extrinsic pathway. The expected data is the data obtained using equation (1). The fold change is then obtained using the ratio between the real value and the expected value (equation 2). The average for the 3 co-culture is then obtained. In light orange values below the limits used for the measurements (Fc>2) and in orange values above the threshold.

GeneID	RPKM data							Expected			Real/Expected (fold change)			Fold change Average
	A211	A211+ch	A283	A283+ch	A258	A258+ch	ch	A211+ch	A283+ch	A258+ch	A211+ch	A283+ch	A258+ch	
ENDOG	2,35	3,23	3,16	3,93	2,82	5,41	4,63	3,33	3,79	3,60	0,97	1,04	1,50	1,17
DIABLO	13,89	16,03	13,40	19,25	12,25	19,48	15,37	14,53	14,25	13,59	1,10	1,35	1,43	1,30
HTRA2	5,51	7,10	8,46	14,04	5,53	9,00	5,93	5,69	7,37	5,70	1,25	1,90	1,58	1,58
CAD	3,15	4,15	3,41	5,97	3,01	4,95	3,12	3,14	3,28	3,06	1,32	1,82	1,62	1,59
AIFM1	3,94	5,09	5,24	7,94	3,25	5,80	3,51	3,75	4,50	3,37	1,36	1,77	1,72	1,62
TNFRSF10A	2,78	5,79	1,67	7,86	1,74	7,15	2,94	2,85	2,22	2,25	2,03	3,55	3,17	2,92
TRAF1	2,32	5,50	3,91	13,29	2,17	7,34	0,83	1,68	2,58	1,59	3,28	5,14	4,61	4,34

Supplemental Table 6: Description of the 86 up-regulated genes using STRING.

**BDKRB1**	*B1 bradykinin receptor; This is a receptor for bradykinin. Could be a factor in chronic pain and inflammation (353 aa)*
**TNFRSF10A**	*Tumor necrosis factor receptor superfamily member 10A; Receptor for the cytotoxic ligand TNFSF10/TRAIL. The adapter molecule FADD recruits caspase-8 to the activated receptor. The resulting death-inducing signaling complex (DISC) performs caspase-8 proteolytic activation which initiates the subsequent cascade of caspases (aspartate-specific cysteine proteases) mediating apoptosis. Promotes the activation of NF-kappa-B; CD molecules (468 aa)*
**RASD1**	*Dexamethasone-induced Ras-related protein 1; Small GTPase. Negatively regulates the transcription regulation activity of the APBB1/FE65-APP complex via its interaction with APBB1/FE65 (By similarity); Belongs to the small GTPase superfamily. RasD family (281 aa)*
**SLC15A3**	*Solute carrier family 15 member 3; Proton oligopeptide cotransporter. Transports free histidine and certain di- and tripeptides (By similarity); Solute carriers (581 aa)*
**OAS3**	*2'-5'-oligoadenylate synthase 3; Interferon-induced, dsRNA-activated antiviral enzyme which plays a critical role in cellular innate antiviral response. In addition, it may also play a role in other cellular processes such as apoptosis, cell growth, differentiation and gene regulation. Synthesizes preferentially dimers of 2'-5'-oligoadenylates (2-5A) from ATP which then bind to the inactive monomeric form of ribonuclease L (RNase L) leading to its dimerization and subsequent activation. Activation of RNase L leads to degradation of cellular as well as viral RNA, resulting in the inhib [...] (1087 aa)*
**HBEGF**	*Proheparin-binding EGF-like growth factor; Growth factor that mediates its effects via EGFR, ERBB2 and ERBB4. Required for normal cardiac valve formation and normal heart function. Promotes smooth muscle cell proliferation. May be involved in macrophage-mediated cellular proliferation. It is mitogenic for fibroblasts, but not endothelial cells. It is able to bind EGF receptor/EGFR with higher affinity than EGF itself and is a far more potent mitogen for smooth muscle cells than EGF. Also acts as a diphtheria toxin receptor (208 aa)*
**EVI2A**	*Protein EVI2A; May complex with itself or/and other proteins within the membrane, to function as part of a cell-surface receptor (259 aa)*
**RARRES3**	*Retinoic acid receptor responder protein 3; Exhibits PLA1/2 activity, catalyzing the calcium- independent hydrolysis of acyl groups in various phosphotidylcholines (PC) and phosphatidylethanolamine (PE). For most substrates, PLA1 activity is much higher than PLA2 activity. N- and O-acylation activity is hardly detectable; HRAS like suppressor family (164 aa)*
**IRAK2**	*Interleukin-1 receptor-associated kinase-like 2; Binds to the IL-1 type I receptor following IL-1 engagement, triggering intracellular signaling cascades leading to transcriptional up-regulation and mRNA stabilization (625 aa)*
**SPX**	*Spexin; Plays a role as a central modulator of cardiovascular and renal function and nociception. Plays also a role in energy metabolism and storage. Inhibits adrenocortical cell proliferation with minor stimulation on corticosteroid release (By similarity) (116 aa)*
**PIK3R3**	*Phosphatidylinositol 3-kinase regulatory subunit gamma; Binds to activated (phosphorylated) protein-tyrosine kinases through its SH2 domain and regulates their kinase activity. During insulin stimulation, it also binds to IRS-1 (461 aa)*
**BIRC3**	*Baculoviral IAP repeat-containing protein 3; Multi-functional protein which regulates not only caspases and apoptosis, but also modulates inflammatory signaling and immunity, mitogenic kinase signaling and cell proliferation, as well as cell invasion and metastasis. Acts as an E3 ubiquitin- protein ligase regulating NF-kappa-B signaling and regulates both canonical and non-canonical NF-kappa-B signaling by acting in opposite directions- acts as a positive regulator of the canonical pathway and suppresses constitutive activation of non-canonical NF-kappa-B signaling. The target proteins [...] (604 aa)*
**IFIH1**	*Interferon-induced helicase C domain-containing protein 1; Innate immune receptor which acts as a cytoplasmic sensor of viral nucleic acids and plays a major role in sensing viral infection and in the activation of a cascade of antiviral responses including the induction of type I interferons and proinflammatory cytokines. Its ligands include mRNA lacking 2'-O- methylation at their 5' cap and long-dsRNA (>1 kb in length). Upon ligand binding it associates with mitochondria antiviral signaling protein (MAVS/IPS1) which activates the IKK-related kinases- TBK1 and IKBKE which phosphorylat [...] (1025 aa)*
**FNIP2**	*Folliculin-interacting protein 2; Acts as a co-chaperone of HSP90AA1. Inhibits the ATPase activity of HSP90AA1 leading to reduction in its chaperone activity. Facilitates the binding of client protein FLCN to HSP90AA1. May play a role in the signal transduction pathway of apoptosis induced by O6-methylguanine- mispaired lesions (By similarity). May be involved in energy and/or nutrient sensing through the AMPK and mTOR signaling pathways. May regulate phosphorylation of RPS6KB1; DENN/MADD domain containing (1114 aa)*
**NPC1**	*Niemann-Pick C1 protein; Intracellular cholesterol transporter which acts in concert with NPC2 and plays an important role in the egress of cholesterol from the endosomal/lysosomal compartment. Both NPC1 and NPC2 function as the cellular 'tag team duo' (TTD) to catalyze the mobilization of cholesterol within the multivesicular environment of the late endosome (LE) to effect egress through the limiting bilayer of the LE. NPC2 binds unesterified cholesterol that has been released from LDLs in the lumen of the late endosome/lysosomes and transfers it to the cholesterol-binding pocket of [...] (1278 aa)*
**NFKBIE**	*NF-kappa-B inhibitor epsilon; Inhibits NF-kappa-B by complexing with and trapping it in the cytoplasm. Inhibits DNA-binding of NF-kappa-B p50-p65 and p50-c-Rel complexes (500 aa)*
**CDKN2B**	*Cyclin-dependent kinase 4 inhibitor B; Interacts strongly with CDK4 and CDK6. Potent inhibitor. Potential effector of TGF-beta induced cell cycle arrest; Belongs to the CDKN2 cyclin-dependent kinase inhibitor family (138 aa)*
**SPINK1**	*Serine protease inhibitor Kazal-type 1; Serine protease inhibitor which exhibits anti-trypsin activity. In the pancreas, protects against trypsin-catalyzed premature activation of zymogens (By similarity); Serine peptidase inhibitors, Kazal type (79 aa)*
**GEM**	*GTP-binding protein GEM; Could be a regulatory protein, possibly participating in receptor-mediated signal transduction at the plasma membrane. Has guanine nucleotide-binding activity but undetectable intrinsic GTPase activity; RGK type GTPase family (296 aa)*
**RRAD**	*GTP-binding protein RAD; May play an important role in cardiac antiarrhythmia via the strong suppression of voltage-gated L-type Ca(2+) currents. Regulates voltage-dependent L-type calcium channel subunit alpha- 1C trafficking to the cell membrane (By similarity). Inhibits cardiac hypertrophy through the calmodulin-dependent kinase II (CaMKII) pathway. Inhibits phosphorylation and activation of CAMK2D; Belongs to the small GTPase superfamily. RGK family (308 aa)*
**HIST1H1E**	*Histone H1.4; Histone H1 protein binds to linker DNA between nucleosomes forming the macromolecular structure known as the chromatin fiber. Histones H1 are necessary for the condensation of nucleosome chains into higher-order structured fibers. Acts also as a regulator of individual gene transcription through chromatin remodeling, nucleosome spacing and DNA methylation (By similarity) (219 aa)*
**EIF4EBP3**	*Eukaryotic translation initiation factor 4E-binding protein 3; Repressor of translation initiation that regulates EIF4E activity by preventing its assembly into the eIF4F complex- hypophosphorylated form competes with EIF4G1/EIF4G3 and strongly binds to EIF4E, leading to repress translation. In contrast, hyperphosphorylated form dissociates from EIF4E, allowing interaction between EIF4G1/EIF4G3 and EIF4E, leading to initiation of translation (100 aa)*
**C10orf2**	*Twinkle protein, mitochondrial; Involved in mitochondrial DNA (mtDNA) metabolism. Could function as an adenine nucleotide-dependent DNA helicase. Function inferred to be critical for lifetime maintenance of mtDNA integrity. In vitro, forms in combination with POLG, a processive replication machinery, which can use double-stranded DNA (dsDNA) as template to synthesize single-stranded DNA (ssDNA) molecules. May be a key regulator of mtDNA copy number in mammals (684 aa)*
**FOSL1**	*Fos-related antigen 1; FOS like 1, AP-1 transcription factor subunit; Belongs to the bZIP family. Fos subfamily (271 aa)*
**PLD6**	*Mitochondrial cardiolipin hydrolase; Endonuclease that plays a critical role in PIWI- interacting RNA (piRNA) biogenesis during spermatogenesis. piRNAs provide essential protection against the activity of mobile genetic elements (By similarity). piRNA-mediated transposon silencing is thus critical for maintaining genome stability, in particular in germline cells when transposons are mobilized as a consequence of wide-spread genomic demethylation (By similarity). Has been proposed to act as a cardiolipin hydrolase to generate phosphatidic acid at mitochondrial surface (By similarity). A [...] (252 aa)*
**MET**	*Hepatocyte growth factor receptor; Receptor tyrosine kinase that transduces signals from the extracellular matrix into the cytoplasm by binding to hepatocyte growth factor/HGF ligand. Regulates many physiological processes including proliferation, scattering, morphogenesis and survival. Ligand binding at the cell surface induces autophosphorylation of MET on its intracellular domain that provides docking sites for downstream signaling molecules. Following activation by ligand, interacts with the PI3-kinase subunit PIK3R1, PLCG1, SRC, GRB2, STAT3 or the adapter GAB1. Recruitment of thes [...] (1408 aa)*
**RPP25**	*Ribonuclease P protein subunit p25; Component of ribonuclease P, a protein complex that generates mature tRNA molecules by cleaving their 5'-ends. Also a component of RNase MRP. This subunit binds to RNA (199 aa)*
**HSPH1**	*Heat shock protein 105 kDa; Acts as a nucleotide-exchange factor (NEF) for chaperone proteins HSPA1A and HSPA1B, promoting the release of ADP from HSPA1A/B thereby triggering client/substrate protein release. Prevents the aggregation of denatured proteins in cells under severe stress, on which the ATP levels decrease markedly. Inhibits HSPA8/HSC70 ATPase and chaperone activities (By similarity); Heat shock 70kDa proteins (858 aa)*
**PHLDA2**	*Pleckstrin homology-like domain family A member 2; Plays a role in regulating placenta growth. May act via its PH domain that competes with other PH domain-containing proteins, thereby preventing their binding to membrane lipids (By similarity); Belongs to the PHLDA2 family (152 aa)*
**SLC7A8**	*Large neutral amino acids transporter small subunit 2; Sodium-independent, high-affinity transport of small and large neutral amino acids such as alanine, serine, threonine, cysteine, phenylalanine, tyrosine, leucine, arginine and tryptophan, when associated with SLC3A2/4F2hc. Acts as an amino acid exchanger. Has higher affinity for L-phenylalanine than LAT1 but lower affinity for glutamine and serine. L-alanine is transported at physiological concentrations. Plays a role in basolateral (re)absorption of neutral amino acids. Involved in the uptake of methylmercury (MeHg) when administe [...] (535 aa)*
**RHBDF2**	*Inactive rhomboid protein 2; Rhomboid protease-like protein which has no protease activity but regulates the secretion of several ligands of the epidermal growth factor receptor. Indirectly activates the epidermal growth factor receptor signaling pathway and may thereby regulate sleep, cell survival, proliferation and migration (By similarity); Belongs to the peptidase S54 family (856 aa)*

**MTSS1**	*Metastasis suppressor protein 1; May be related to cancer progression or tumor metastasis in a variety of organ sites, most likely through an interaction with the actin cytoskeleton; I-BAR domain containing (759 aa)*
**LSMEM1**	*Leucine rich single-pass membrane protein 1 (131 aa)*
**CSF1**	*Macrophage colony-stimulating factor 1; Cytokine that plays an essential role in the regulation of survival, proliferation and differentiation of hematopoietic precursor cells, especially mononuclear phagocytes, such as macrophages and monocytes. Promotes the release of proinflammatory chemokines, and thereby plays an important role in innate immunity and in inflammatory processes. Plays an important role in the regulation of osteoclast proliferation and differentiation, the regulation of bone resorption, and is required for normal bone development. Required for normal male and female [...] (554 aa)*
**C5orf56**	*Uncharacterized protein C5orf56; Chromosome 5 open reading frame 56 (126 aa)*
**B7RP1**	*Inducible T-cell co-stimulator ligand; Ligand for the T-cell-specific cell surface receptor ICOS. Acts as a costimulatory signal for T-cell proliferation and cytokine secretion; induces also B-cell proliferation and differentiation into plasma cells. Could play an important role in mediating local tissue responses to inflammatory conditions, as well as in modulating the secondary immune response by co- stimulating memory T-cell function (By similarity); Belongs to the immunoglobulin superfamily. BTN/MOG family (309 aa)*
**LRRC37B**	*Leucine-rich repeat-containing protein 37B; Leucine rich repeat containing 37B (947 aa)*
**NCR3LG1**	*Natural cytotoxicity triggering receptor 3 ligand 1; Triggers NCR3-dependent natural killer cell activation; C1-set domain containing (454 aa)*
**ATF3**	*Cyclic AMP-dependent transcription factor ATF-3; This protein binds the cAMP response element (CRE) (consensus- 5'-GTGACGT[AC][AG]-3'), a sequence present in many viral and cellular promoters. Represses transcription from promoters with ATF sites. It may repress transcription by stabilizing the binding of inhibitory cofactors at the promoter. Isoform 2 activates transcription presumably by sequestering inhibitory cofactors away from the promoters; Basic leucine zipper proteins (181 aa)*
**APOL3**	*Apolipoprotein L3; May affect the movement of lipids in the cytoplasm or allow the binding of lipids to organelles; Apolipoproteins (402 aa)*
**SVIP**	*Small VCP interacting protein; Belongs to the SVIP family (77 aa)*
**TAP1**	*Antigen peptide transporter 1; Involved in the transport of antigens from the cytoplasm to the endoplasmic reticulum for association with MHC class I molecules. Also acts as a molecular scaffold for the final stage of MHC class I folding, namely the binding of peptide. Nascent MHC class I molecules associate with TAP via tapasin. Inhibited by the covalent attachment of herpes simplex virus ICP47 protein, which blocks the peptide-binding site of TAP. Inhibited by human cytomegalovirus US6 glycoprotein, which binds to the lumenal side of the TAP complex and inhibits peptide translocation [...] (808 aa)*
**HIST1H2AG**	*Histone cluster 1 H2A family member g; Core component of nucleosome. Nucleosomes wrap and compact DNA into chromatin, limiting DNA accessibility to the cellular machineries which require DNA as a template. Histones thereby play a central role in transcription regulation, DNA repair, DNA replication and chromosomal stability. DNA accessibility is regulated via a complex set of post-translational modifications of histones, also called histone code, and nucleosome remodeling (130 aa)*
**HIST3H2A**	*Histone H2A type 3; Core component of nucleosome. Nucleosomes wrap and compact DNA into chromatin, limiting DNA accessibility to the cellular machineries which require DNA as a template. Histones thereby play a central role in transcription regulation, DNA repair, DNA replication and chromosomal stability. DNA accessibility is regulated via a complex set of post-translational modifications of histones, also called histone code, and nucleosome remodeling (130 aa)*
**NCF2**	*Neutrophil cytosol factor 2; NCF2, NCF1, and a membrane bound cytochrome b558 are required for activation of the latent NADPH oxidase (necessary for superoxide production); Tetratricopeptide repeat domain containing (526 aa)*
**RGS16**	*Regulator of G-protein signaling 16; Regulates G protein-coupled receptor signaling cascades. Inhibits signal transduction by increasing the GTPase activity of G protein alpha subunits, thereby driving them into their inactive GDP-bound form. Plays an important role in the phototransduction cascade by regulating the lifetime and effective concentration of activated transducin alpha. May regulate extra and intracellular mitogenic signals (By similarity) (202 aa)*
**PAPPA2**	*Pappalysin-2; Metalloproteinase which specifically cleaves insulin- like growth factor binding protein (IGFBP)-5 at the '163-Ser-|- Lys-164' bond. Shows limited proteolysis toward IGFBP-3; Belongs to the peptidase M43B family (1791 aa)*
**SGK1**	*Serine/threonine-protein kinase Sgk1; Serine/threonine-protein kinase which is involved in the regulation of a wide variety of ion channels, membrane transporters, cellular enzymes, transcription factors, neuronal excitability, cell growth, proliferation, survival, migration and apoptosis. Plays an important role in cellular stress response. Contributes to regulation of renal Na(+) retention, renal K(+) elimination, salt appetite, gastric acid secretion, intestinal Na(+)/H(+) exchange and nutrient transport, insulin-dependent salt sensitivity of blood pressure, salt sensitivity of peri [...] (526 aa)*
**GBP1**	*Guanylate-binding protein 1; Hydrolyzes GTP to GMP in 2 consecutive cleavage reactions. Exhibits antiviral activity against influenza virus. Promote oxidative killing and deliver antimicrobial peptides to autophagolysosomes, providing broad host protection against different pathogen classes (592 aa)*
**DIRAS3**	*GTP-binding protein Di-Ras3; RAS type GTPase family (229 aa)*
**TSPAN1**	*Tetraspanin-1; Tetraspanin 1; Tetraspanins (241 aa)*
**MAFB**	*Transcription factor MafB; Acts as a transcriptional activator or repressor. Plays a pivotal role in regulating lineage- specific hematopoiesis by repressing ETS1-mediated transcription of erythroid-specific genes in myeloid cells. Required for monocytic, macrophage, osteoclast, podocyte and islet beta cell differentiation. Involved in renal tubule survival and F4/80 maturation. Activates the insulin and glucagon promoters. Together with PAX6, transactivates weakly the glucagon gene promoter through the G1 element. SUMO modification controls its transcriptional activity and ability to [...] (323 aa)*
**FAM167B**	*Protein FAM167B; Family with sequence similarity 167 member B; Belongs to the FAM167 (SEC) family (163 aa)*
**TRAF1**	*TNF receptor-associated factor 1; Adapter molecule that regulates the activation of NF- kappa-B and JNK. Plays a role in the regulation of cell survival and apoptosis. The heterotrimer formed by TRAF1 and TRAF2 is part of a E3 ubiquitin-protein ligase complex that promotes ubiquitination of target proteins, such as MAP3K14. The TRAF1/TRAF2 complex recruits the antiapoptotic E3 protein- ubiquitin ligases BIRC2 and BIRC3 to TNFRSF1B/TNFR2; TNF receptor associated factors (416 aa)*
**TMEM38B**	*Trimeric intracellular cation channel type B; Monovalent cation channel required for maintenance of rapid intracellular calcium release. May act as a potassium counter-ion channel that functions in synchronization with calcium release from intracellular stores; Belongs to the TMEM38 family (291 aa)*
**PSMB9**	*Proteasome subunit beta type-9; The proteasome is a multicatalytic proteinase complex which is characterized by its ability to cleave peptides with Arg, Phe, Tyr, Leu, and Glu adjacent to the leaving group at neutral or slightly basic pH. The proteasome has an ATP-dependent proteolytic activity. This subunit is involved in antigen processing to generate class I binding peptides. Replacement of PSMB6 by PSMB9 increases the capacity of the immunoproteasome to cleave model peptides after hydrophobic and basic residues (219 aa)*
**PSMB8**	*Proteasome subunit beta type-8; The proteasome is a multicatalytic proteinase complex which is characterized by its ability to cleave peptides with Arg, Phe, Tyr, Leu, and Glu adjacent to the leaving group at neutral or slightly basic pH. The proteasome has an ATP-dependent proteolytic activity. This subunit is involved in antigen processing to generate class I binding peptides. Replacement of PSMB5 by PSMB8 increases the capacity of the immunoproteasome to cleave model peptides after hydrophobic and basic residues. Acts as a major component of interferon gamma-induced sensitivity. Pla [...] (276 aa)*
**TAP2**	*Antigen peptide transporter 2; Involved in the transport of antigens from the cytoplasm to the endoplasmic reticulum for association with MHC class I molecules. Also acts as a molecular scaffold for the final stage of MHC class I folding, namely the binding of peptide. Nascent MHC class I molecules associate with TAP via tapasin. Inhibited by the covalent attachment of herpes simplex virus ICP47 protein, which blocks the peptide-binding site of TAP. Inhibited by human cytomegalovirus US6 glycoprotein, which binds to the lumenal side of the TAP complex and inhibits peptide translocation [...] (653 aa)*
**NINJ1**	*Ninjurin-1; Homophilic cell adhesion molecule that promotes axonal growth. May play a role in nerve regeneration and in the formation and function of other tissues. Cell adhesion requires divalent cations (152 aa)*

**HSPA1B**	*Heat shock protein family A member 1B (641 aa)*
**RND3**	*Rho-related GTP-binding protein RhoE; Binds GTP but lacks intrinsic GTPase activity and is resistant to Rho-specific GTPase-activating proteins; Rho family GTPases (244 aa)*
**CKS2**	*Cyclin-dependent kinases regulatory subunit 2; Binds to the catalytic subunit of the cyclin dependent kinases and is essential for their biological function (79 aa)*
**KIAA1217**	*Sickle tail protein homolog; Required for normal development of intervertebral disks (1943 aa)*
**THBD**	*Thrombomodulin; Thrombomodulin is a specific endothelial cell receptor that forms a 1-1 stoichiometric complex with thrombin. This complex is responsible for the conversion of protein C to the activated protein C (protein Ca). Once evolved, protein Ca scissions the activated cofactors of the coagulation mechanism, factor Va and factor VIIIa, and thereby reduces the amount of thrombin generated; C-type lectin domain containing (575 aa)*
**SLC3A2**	*4F2 cell-surface antigen heavy chain; Required for the function of light chain amino-acid transporters. Involved in sodium-independent, high-affinity transport of large neutral amino acids such as phenylalanine, tyrosine, leucine, arginine and tryptophan. Involved in guiding and targeting of LAT1 and LAT2 to the plasma membrane. When associated with SLC7A6 or SLC7A7 acts as an arginine/glutamine exchanger, following an antiport mechanism for amino acid transport, influencing arginine release in exchange for extracellular amino acids. Plays a role in nitric oxide synthesis in human umbi [...] (631 aa)*
**BMP2**	*Bone morphogenetic protein 2; Induces cartilage and bone formation. Stimulates the differentiation of myoblasts into osteoblasts via the EIF2AK3-EIF2A- ATF4 pathway. BMP2 activation of EIF2AK3 stimulates phosphorylation of EIF2A which leads to increased expression of ATF4 which plays a central role in osteoblast differentiation. In addition stimulates TMEM119, which upregulates the expression of ATF4; Belongs to the TGF-beta family (396 aa)*
**ISG15**	*Ubiquitin-like protein ISG15; Ubiquitin-like protein which plays a key role in the innate immune response to viral infection either via its conjugation to a target protein (ISGylation) or via its action as a free or unconjugated protein. ISGylation involves a cascade of enzymatic reactions involving E1, E2, and E3 enzymes which catalyze the conjugation of ISG15 to a lysine residue in the target protein. Its target proteins include IFIT1, MX1/MxA, PPM1B, UBE2L6, UBA7, CHMP5, CHMP2A, CHMP4B and CHMP6. Can also isgylate- EIF2AK2/PKR which results in its activation, DDX58/RIG-I which inhib [...] (165 aa)*
**AKR1C1**	*Aldo-keto reductase family 1 member C1; Converts progesterone to its inactive form, 20-alpha- dihydroxyprogesterone (20-alpha-OHP). In the liver and intestine, may have a role in the transport of bile. May have a role in monitoring the intrahepatic bile acid concentration. Has a low bile-binding ability. May play a role in myelin formation; Belongs to the aldo/keto reductase family (323 aa)*
**SQSTM1**	*Sequestosome-1; Autophagy receptor that interacts directly with both the cargo to become degraded and an autophagy modifier of the MAP1 LC3 family. Along with WDFY3, involved in the formation and autophagic degradation of cytoplasmic ubiquitin-containing inclusions (p62 bodies, ALIS/aggresome-like induced structures). Along with SQSTM1, required to recruit ubiquitinated proteins to PML bodies in the nucleus. May regulate the activation of NFKB1 by TNF-alpha, nerve growth factor (NGF) and interleukin-1. May play a role in titin/TTN downstream signaling in muscle cells. May regulate sign [...] (440 aa)*
**KCNJ14**	*ATP-sensitive inward rectifier potassium channel 14; Inward rectifier potassium channels are characterized by a greater tendency to allow potassium to flow into the cell rather than out of it. Their voltage dependence is regulated by the concentration of extracellular potassium; as external potassium is raised, the voltage range of the channel opening shifts to more positive voltages. The inward rectification is mainly due to the blockage of outward current by internal magnesium. KCNJ14 gives rise to low-conductance channels with a low affinity to the channel blockers Barium and Cesium [...] (436 aa)*
**GAL3ST1**	*Galactosylceramide sulfotransferase; Catalyzes the sulfation of membrane glycolipids. Seems to prefer beta-glycosides at the non-reducing termini of sugar chains attached to a lipid moiety. Catalyzes the synthesis of galactosylceramide sulfate (sulfatide), a major lipid component of the myelin sheath and of monogalactosylalkylacylglycerol sulfate (seminolipid), present in spermatocytes (By similarity). Also acts on lactosylceramide, galactosyl 1-alkyl-2-sn-glycerol and galactosyl diacylglycerol (in vitro); Belongs to the galactose-3-O-sulfotransferase family (423 aa)*
**RGS14**	*Regulator of G-protein signaling 14; Regulates G protein-coupled receptor signaling cascades. Inhibits signal transduction by increasing the GTPase activity of G protein alpha subunits, thereby driving them into their inactive GDP-bound form. Besides, modulates signal transduction via G protein alpha subunits by functioning as a GDP-dissociation inhibitor (GDI). Has GDI activity on G(i) alpha subunits GNAI1 and GNAI3, but not on GNAI2 and G(o) alpha subunit GNAO1. Has GAP activity on GNAI0, GNAI2 and GNAI3. May act as a scaffold integrating G protein and Ras/Raf MAPkinase signaling pat [...] (566 aa)*
**HMGA1**	*High mobility group protein HMG-I/HMG-Y; HMG-I/Y bind preferentially to the minor groove of A+T rich regions in double-stranded DNA. It is suggested that these proteins could function in nucleosome phasing and in the 3'-end processing of mRNA transcripts. They are also involved in the transcription regulation of genes containing, or in close proximity to A+T-rich regions; Canonical high mobility group (107 aa)*
**SPATA24**	*Spermatogenesis-associated protein 24; Binds DNA with high affinity but does not bind to TATA boxes. Synergises with GMNN and TBP in activation of TATA box-containing promoters and with GMNN and TBPL1 in activation of the NF1 TATA-less promoter. May play a role in cytoplasm movement and removal during spermiogenesis (By similarity) (205 aa)*
**C6orf1**	*Uncharacterized protein SMIM29; Chromosome 6 open reading frame 1 (159 aa)*
**CLDN23**	*Claudin-23; Plays a major role in tight junction-specific obliteration of the intercellular space, through calcium- independent cell-adhesion activity; Claudins (292 aa)*
**GPR68**	*Ovarian cancer G-protein coupled receptor 1; Proton-sensing receptor involved in pH homeostasis. May represents an osteoblastic pH sensor regulating cell-mediated responses to acidosis in bone. Mediates its action by association with G proteins that stimulates inositol phosphate (IP) production or Ca(2+) mobilization. The receptor is almost silent at pH 7.8 but fully activated at pH 6.8. Function also as a metastasis suppressor gene in prostate cancer (By similarity); Belongs to the G-protein coupled receptor 1 family (365 aa)*
**HIST1H2BG**	*Histone cluster 1 H2B family member g; Core component of nucleosome. Nucleosomes wrap and compact DNA into chromatin, limiting DNA accessibility to the cellular machineries which require DNA as a template. Histones thereby play a central role in transcription regulation, DNA repair, DNA replication and chromosomal stability. DNA accessibility is regulated via a complex set of post-translational modifications of histones, also called histone code, and nucleosome remodeling (126 aa)*
**SOD2**	*Superoxide dismutase [Mn], mitochondrial; Destroys superoxide anion radicals which are normally produced within the cells and which are toxic to biological systems (222 aa)*
**TRIM16L**	*Tripartite motif-containing protein 16-like protein; Tripartite motif containing 16 like; Belongs to the TRIM/RBCC family (348 aa)*
**MIF4GD**	*MIF4G domain-containing protein; MIF4G domain containing (263 aa)*
**SEC11C**	*Signal peptidase complex catalytic subunit SEC11C; Component of the microsomal signal peptidase complex which removes signal peptides from nascent proteins as they are translocated into the lumen of the endoplasmic reticulum (192 aa)*
**IL4I1**	*L-amino-acid oxidase; Lysosomal L-amino-acid oxidase with highest specific activity with phenylalanine. May play a role in lysosomal antigen processing and presentation (By similarity); Belongs to the flavin monoamine oxidase family. FIG1 subfamily (589 aa)*
**PLA2G4C**	*Cytosolic phospholipase A2 gamma; Has a preference for arachidonic acid at the sn-2 position of phosphatidylcholine as compared with palmitic acid; Phospholipases (551 aa)*
**FOLR3**	*Folate receptor gamma; Binds to folate and reduced folic acid derivatives and mediates delivery of 5-methyltetrahydrofolate to the interior of cells. Isoform Short does not bind folate (245 aa)*
**HPCAL1**	*Hippocalcin-like protein 1; May be involved in the calcium-dependent regulation of rhodopsin phosphorylation; Belongs to the recoverin family (193 aa)*

~~Supplementary~~ Supplemental Table 7: RPKM values of 8 proliferation genes The expected value of the co-culture is calculated considering the ratio determined with the male markers (43% chondrocytes & 57% MSCs). The value is then compared to the actual value of the co-culture and an average is obtained indicating the fold-change of the real value compared to the expected.

GeneID	A211	A211+ch	A283	A283+ch	A258	A258+ch	ch		211A(57%)+ ch(43%)	283A(57%)+ ch(43%)	258A(57%)+ ch(43%)		A211+ch /211A(57%) + ch(43%)	A283+ch /283A(57%) + ch(43%)	A258+ch /258A(57%) + ch(43%)		average
MKI67	0,86	0,57	0,09	0,26	0,26	0,25	0,15		0,56	0,11	0,22		1,03	2,27	1,18		1,49
MYC	17,18	16,20	12,93	13,07	15,77	15,08	6,41		12,55	10,13	11,75		1,29	1,29	1,28		1,29
PCNA	29,13	35,53	20,49	49,41	18,46	37,86	27,09		28,25	23,33	22,17		1,26	2,12	1,71		1,69
MCM2	3,87	3,68	2,76	3,97	2,61	5,99	3,73		3,81	3,18	3,09		0,97	1,25	1,94		1,38
MCM7	15,88	16,74	11,41	14,44	10,58	16,93	10,36		13,51	10,96	10,49		1,24	1,32	1,61		1,39
CCND1	53,98	63,68	63,10	111,92	159,83	112,17	41,71		48,70	53,90	109,04		1,31	2,08	1,03		1,47
CCNE1	1,39	1,02	1,10	1,74	0,76	1,34	0,59		1,05	0,89	0,69		0,97	1,96	1,95		1,62
CCNB1	2,63	1,91	1,21	2,01	1,20	2,95	0,77		1,83	1,02	1,02		1,04	1,97	2,91		1,97

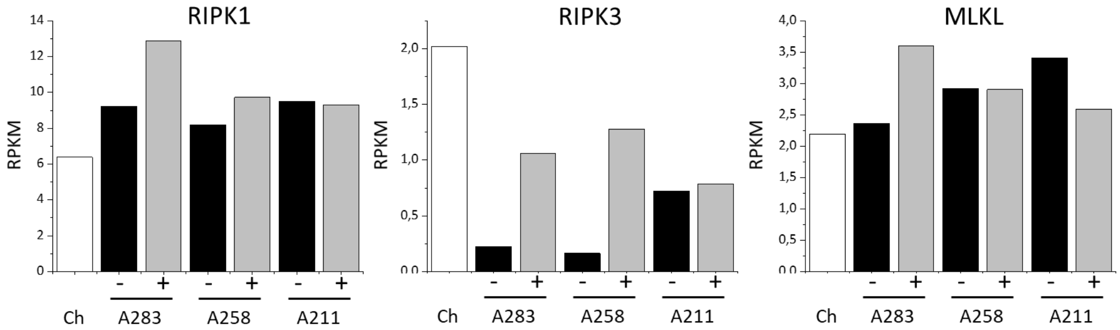

Supplementary Figure 4: RPKM values of 3 key regulatory markers of the regulated necrosis.

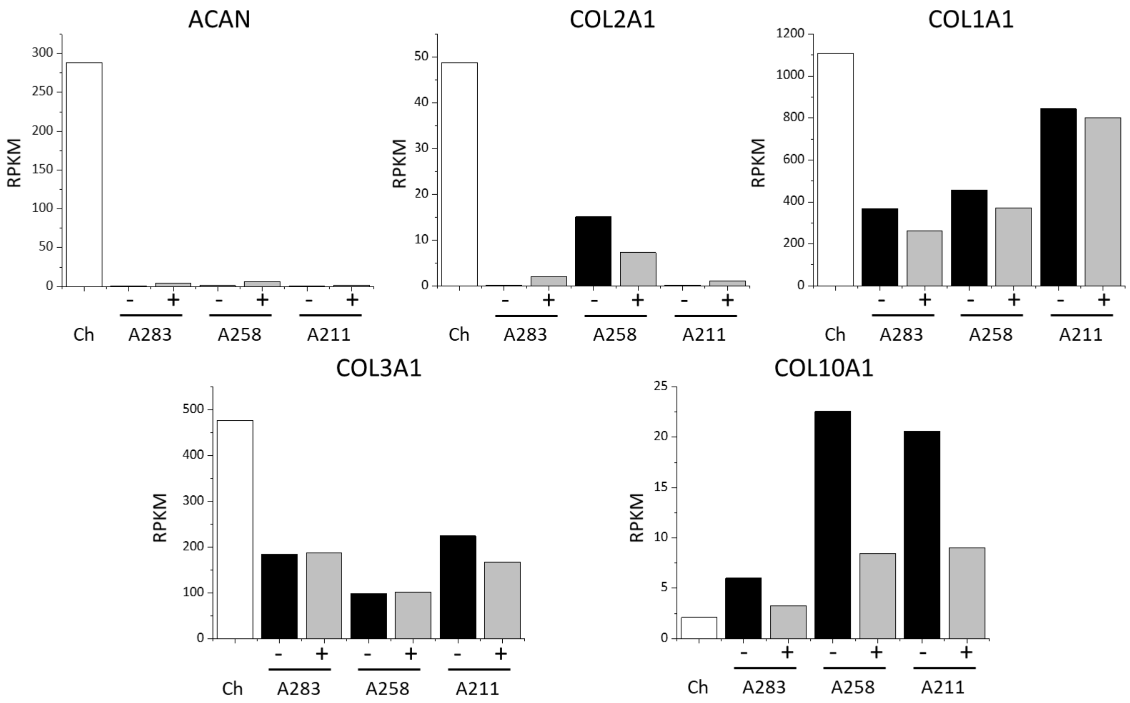

Supplementary Figure 5: Comparison between RPKM values of chondrocytes markers. The first raw shows chondrogenic markers (ACAN,COL2A1, COL1A1). The second raw indicate osteoarthritic markers (COL3A1, COL10A1).

Supplemental References

1. Feng M, Xiong G, Cao Z, Yang G, Zheng S, Qiu J, et al. LAT2 regulates glutamine-dependent mTOR activation to promote glycolysis and chemoresistance in pancreatic cancer. J Exp Clin Cancer Res 2018; 37: 274.
2. Feral CC, Nishiya N, Fenczik CA, Stuhlmann H, Slepak M, Ginsberg MH. CD98hc (SLC3A2) mediates integrin signaling. Proc Natl Acad Sci U S A 2005; 102: 355-360.
3. He W, Wang Q, Xu J, Xu X, Padilla MT, Ren G, et al. Attenuation of TNFSF10/TRAIL-induced apoptosis by an autophagic survival pathway involving TRAF2- and RIPK1/RIP1-mediated MAPK8/JNK activation. Autophagy 2012; 8: 1811-1821.
4. Tattoli I, Philpott DJ, Girardin SE. The bacterial and cellular determinants controlling the recruitment of mTOR to the Salmonella-containing vacuole. Biol Open 2012; 1: 1215-1225.
5. Schuster AT, Homer CR, Kemp JR, Nickerson KP, Deutschman E, Kim Y, et al. Chromosome-associated protein D3 promotes bacterial clearance in human intestinal epithelial cells by repressing expression of amino acid transporters. Gastroenterology 2015; 148: 1405-1416 e1403.
6. Hamada M, Nakamura M, Tran MT, Moriguchi T, Hong C, Ohsumi T, et al. MafB promotes atherosclerosis by inhibiting foam-cell apoptosis. Nat Commun 2014; 5: 3147.
7. Lin H, Li HF, Chen HH, Lai PF, Juan SH, Chen JJ, et al. Activating transcription factor 3 protects against pressure-overload heart failure via the autophagy molecule Beclin-1 pathway. Mol Pharmacol 2014; 85: 682-691.
8. Choi SR, Chung BY, Kim SW, Kim CD, Yun WJ, Lee MW, et al. Activation of autophagic pathways is related to growth inhibition and senescence in cutaneous squamous cell carcinoma. Exp Dermatol 2014; 23: 718-724.
9. Sood V, Sharma KB, Gupta V, Saha D, Dhapola P, Sharma M, et al. ATF3 negatively regulates cellular antiviral signaling and autophagy in the absence of type I interferons. Sci Rep 2017; 7: 8789.
10. Hartman MG, Lu D, Kim ML, Kociba GJ, Shukri T, Buteau J, et al. Role for activating transcription factor 3 in stress-induced beta-cell apoptosis. Mol Cell Biol 2004; 24: 5721-5732.
11. Nakagomi S, Suzuki Y, Namikawa K, Kiryu-Seo S, Kiyama H. Expression of the activating transcription factor 3 prevents c-Jun N-terminal kinase-induced neuronal death by promoting heat shock protein 27 expression and Akt activation. J Neurosci 2003; 23: 5187-5196.
12. Nobori K, Ito H, Tamamori-Adachi M, Adachi S, Ono Y, Kawauchi J, et al. ATF3 inhibits doxorubicin-induced apoptosis in cardiac myocytes: a novel cardioprotective role of ATF3. J Mol Cell Cardiol 2002; 34: 1387-1397.
13. Lu D, Wolfgang CD, Hai T. Activating transcription factor 3, a stress-inducible gene, suppresses Ras-stimulated tumorigenesis. J Biol Chem 2006; 281: 10473-10481.
14. Yin X, Dewille JW, Hai T. A potential dichotomous role of ATF3, an adaptive-response gene, in cancer development. Oncogene 2008; 27: 2118-2127.
15. Foo LC, Allen NJ, Bushong EA, Ventura PB, Chung WS, Zhou L, et al. Development of a method for the purification and culture of rodent astrocytes. Neuron 2011; 71: 799-811.
16. Armant DR, Kilburn BA, Petkova A, Edwin SS, Duniec-Dmuchowski ZM, Edwards HJ, et al. Human trophoblast survival at low oxygen concentrations requires metalloproteinase-mediated shedding of heparin-binding EGF-like growth factor. Development 2006; 133: 751-759.
17. Imudia AN, Kilburn BA, Petkova A, Edwin SS, Romero R, Armant DR. Expression of heparin-binding EGF-like growth factor in term chorionic villous explants and its role in trophoblast survival. Placenta 2008; 29: 784-789.
18. Leach RE, Kilburn BA, Petkova A, Romero R, Armant DR. Diminished survival of human cytotrophoblast cells exposed to hypoxia/reoxygenation injury and associated reduction of heparin-binding epidermal growth factor-like growth factor. Am J Obstet Gynecol 2008; 198: 471 e471-477; discussion 471 e477-478.

19. Zhou J, Yang J, Fan X, Hu S, Zhou F, Dong J, et al. Chaperone-mediated autophagy regulates proliferation by targeting RND3 in gastric cancer. Autophagy 2016; 12: 515-528.
20. Ongusaha PP, Kim HG, Boswell SA, Ridley AJ, Der CJ, Dotto GP, et al. RhoE is a pro-survival p53 target gene that inhibits ROCK I-mediated apoptosis in response to genotoxic stress. Curr Biol 2006; 16: 2466-2472.
21. Yue X, Yang X, Lin X, Yang T, Yi X, Dai Y, et al. Rnd3 haploinsufficient mice are predisposed to hemodynamic stress and develop apoptotic cardiomyopathy with heart failure. Cell Death Dis 2014; 5: e1284.
22. Boswell SA, Ongusaha PP, Nghiem P, Lee SW. The protective role of a small GTPase RhoE against UVB-induced DNA damage in keratinocytes. J Biol Chem 2007; 282: 4850-4858.
23. Wang Y, Liu G, Ren L, Wang K, Liu A. Long non-coding RNA TUG1 recruits miR29c3p from its target gene RGS1 to promote proliferation and metastasis of melanoma cells. Int J Oncol 2019; 54: 1317-1326.
24. Fang J, Liu X, Bolanos L, Barker B, Rigolino C, Cortelezzi A, et al. A calcium- and calpain-dependent pathway determines the response to lenalidomide in myelodysplastic syndromes. Nat Med 2016; 22: 727-734.
25. Kakoki M, McGarrah RW, Kim HS, Smithies O. Bradykinin B1 and B2 receptors both have protective roles in renal ischemia/reperfusion injury. Proc Natl Acad Sci U S A 2007; 104: 7576-7581.
26. Zhang C, Lu J, Liu B, Cui Q, Wang Y. Primate-specific miR-603 is implicated in the risk and pathogenesis of Alzheimer's disease. Aging (Albany NY) 2016; 8: 272-290.
27. Panda PK, Naik PP, Praharaj PP, Meher BR, Gupta PK, Verma RS, et al. Abrus agglutinin stimulates BMP-2-dependent differentiation through autophagic degradation of beta-catenin in colon cancer stem cells. Mol Carcinog 2018; 57: 664-677.
28. Cao Y, Yang W, Tyler MA, Gao X, Duan C, Kim SO, et al. Noggin attenuates cerulein-induced acute pancreatitis and impaired autophagy. Pancreas 2013; 42: 301-307.
29. Hallahan AR, Pritchard JI, Chandraratna RA, Ellenbogen RG, Geyer JR, Overland RP, et al. BMP-2 mediates retinoid-induced apoptosis in medulloblastoma cells through a paracrine effect. Nat Med 2003; 9: 1033-1038.
30. Kawamura C, Kizaki M, Yamato K, Uchida H, Fukuchi Y, Hattori Y, et al. Bone morphogenetic protein-2 induces apoptosis in human myeloma cells with modulation of STAT3. Blood 2000; 96: 2005-2011.
31. Kim HK, Oxendine I, Kamiya N. High-concentration of BMP2 reduces cell proliferation and increases apoptosis via DKK1 and SOST in human primary periosteal cells. Bone 2013; 54: 141-150.
32. Hyzy SL, Olivares-Navarrete R, Schwartz Z, Boyan BD. BMP2 induces osteoblast apoptosis in a maturation state and noggin-dependent manner. J Cell Biochem 2012; 113: 3236-3245.
33. Suchanski J, Grzegrzolka J, Owczarek T, Pasikowski P, Piotrowska A, Kocbach B, et al. Sulfatide decreases the resistance to stress-induced apoptosis and increases P-selectin-mediated adhesion: a two-edged sword in breast cancer progression. Breast Cancer Res 2018; 20: 133.
34. Jin F, Qiao C, Luan N, Li H. Lentivirus-mediated PHLDA2 overexpression inhibits trophoblast proliferation, migration and invasion, and induces apoptosis. Int J Mol Med 2016; 37: 949-957.
35. Lee MP, Feinberg AP. Genomic imprinting of a human apoptosis gene homologue, TSSC3. Cancer Res 1998; 58: 1052-1056.
36. Dai H, Huang Y, Li Y, Meng G, Wang Y, Guo QN. TSSC3 overexpression associates with growth inhibition, apoptosis induction and enhances chemotherapeutic effects in human osteosarcoma. Carcinogenesis 2012; 33: 30-40.
37. Huang Y, Dai H, Guo QN. TSSC3 overexpression reduces stemness and induces apoptosis of osteosarcoma tumor-initiating cells. Apoptosis 2012; 17: 749-761.
38. Zhao GS, Gao ZR, Zhang Q, Tang XF, Lv YF, Zhang ZS, et al. TSSC3 promotes autophagy via inactivating the Src-mediated PI3K/Akt/mTOR pathway to suppress tumorigenesis and metastasis in osteosarcoma, and predicts a favorable prognosis. J Exp Clin Cancer Res 2018; 37: 188.
39. Qiu J, Li X, He Y, Sun D, Li W, Xin Y. Distinct subgroup of the Ras family member 3 (DIRAS3) expression impairs metastasis and induces autophagy of gastric cancer cells in mice. J Cancer Res Clin Oncol 2018; 144: 1869-1886.

40. Zhuo C, Jiang R, Lin X, Shao M. LncRNA H19 inhibits autophagy by epigenetically silencing of DIRAS3 in diabetic cardiomyopathy. Oncotarget 2017; 8: 1429-1437.
41. Ejaz A, Mitterberger MC, Lu Z, Mattesich M, Zwierzina ME, Horl S, et al. Weight Loss Upregulates the Small GTPase DIRAS3 in Human White Adipose Progenitor Cells, Which Negatively Regulates Adipogenesis and Activates Autophagy via Akt-mTOR Inhibition. EBioMedicine 2016; 6: 149-161.
42. Lu Z, Baquero MT, Yang H, Yang M, Reger AS, Kim C, et al. DIRAS3 regulates the autophagosome initiation complex in dormant ovarian cancer cells. Autophagy 2014; 10: 1071-1092.
43. Baljuls A, Beck M, Oenel A, Robubi A, Kroschewski R, Hekman M, et al. The tumor suppressor DiRas3 forms a complex with H-Ras and C-RAF proteins and regulates localization, dimerization, and kinase activity of C-RAF. J Biol Chem 2012; 287: 23128-23140.
44. Conte A, Paladino S, Bianco G, Fasano D, Gerlini R, Tornincasa M, et al. High mobility group A1 protein modulates autophagy in cancer cells. Cell Death Differ 2017; 24: 1948-1962.
45. Takaha N, Sowa Y, Takeuchi I, Hongo F, Kawauchi A, Miki T. Expression and role of HMGA1 in renal cell carcinoma. J Urol 2012; 187: 2215-2222.
46. Akhter MZ, Rajeswari MR. Triplex forming oligonucleotides targeted to hmga1 selectively inhibit its expression and induce apoptosis in human cervical cancer. J Biomol Struct Dyn 2017; 35: 689-703.
47. Kratzmeier M, Albig W, Meergans T, Doenecke D. Changes in the protein pattern of H1 histones associated with apoptotic DNA fragmentation. Biochem J 1999; 337 (Pt 2): 319-327.
48. Happel N, Doenecke D, Sekeri-Pataryas KE, Sourlingas TG. H1 histone subtype constitution and phosphorylation state of the ageing cell system of human peripheral blood lymphocytes. Exp Gerontol 2008; 43: 184-199.
49. Lutz J, Lu R, Strobl M, Huang H, Deng M, Wang M, et al. ICOS/B7RP-1 interference in mouse kidney transplantation. Transplantation 2007; 84: 223-230.
50. Jiang T, Wu W, Zhang H, Zhang X, Zhang D, Wang Q, et al. High expression of B7-H6 in human glioma tissues promotes tumor progression. Oncotarget 2017; 8: 37435-37447.
51. Zhang B, Sun J, Yao X, Li J, Tu Y, Yao F, et al. Knockdown of B7H6 inhibits tumor progression in triple-negative breast cancer. Oncol Lett 2018; 16: 91-96.
52. Yamagishi N, Ishihara K, Saito Y, Hatayama T. Hsp105 family proteins suppress staurosporine-induced apoptosis by inhibiting the translocation of Bax to mitochondria in HeLa cells. Exp Cell Res 2006; 312: 3215-3223.
53. Zappasodi R, Bongarzone I, Ghedini GC, Castagnoli L, Cabras AD, Messina A, et al. Serological identification of HSP105 as a novel non-Hodgkin lymphoma therapeutic target. Blood 2011; 118: 4421-4430.
54. Hosaka S, Nakatsura T, Tsukamoto H, Hatayama T, Baba H, Nishimura Y. Synthetic small interfering RNA targeting heat shock protein 105 induces apoptosis of various cancer cells both in vitro and in vivo. Cancer Sci 2006; 97: 623-632.
55. Hatayama T, Yamagishi N, Minobe E, Sakai K. Role of hsp105 in protection against stress-induced apoptosis in neuronal PC12 cells. Biochem Biophys Res Commun 2001; 288: 528-534.
56. Yamagishi N, Ishihara K, Saito Y, Hatayama T. Hsp105alpha enhances stress-induced apoptosis but not necrosis in mouse embryonal f9 cells. J Biochem 2002; 132: 271-278.
57. Ko SK, Kim J, Na DC, Park S, Park SH, Hyun JY, et al. A small molecule inhibitor of ATPase activity of HSP70 induces apoptosis and has antitumor activities. Chem Biol 2015; 22: 391-403.
58. Kumar S, Stokes J, 3rd, Singh UP, Scissum Gunn K, Acharya A, Manne U, et al. Targeting Hsp70: A possible therapy for cancer. Cancer Lett 2016; 374: 156-166.
59. Huang WJ, Xia LM, Zhu F, Huang B, Zhou C, Zhu HF, et al. Transcriptional upregulation of HSP70-2 by HIF-1 in cancer cells in response to hypoxia. Int J Cancer 2009; 124: 298-305.
60. Kondrikov D, Fulton D, Dong Z, Su Y. Heat Shock Protein 70 Prevents Hyperoxia-Induced Disruption of Lung Endothelial Barrier via Caspase-Dependent and AIF-Dependent Pathways. PLoS One 2015; 10: e0129343.
61. Mustafa N, Ting Lee JX, Adina Nee HF, Bi C, Chung TH, Hart S, et al. VS-5584 mediates potent anti-myeloma activity via the upregulation of a class II tumor suppressor gene, RARRES3 and the activation of Bim. Oncotarget 2017; 8: 101847-101864.

62. Lotz K, Kellner T, Heitmann M, Nazarenko I, Noske A, Malek A, et al. Suppression of the TIG3 tumor suppressor gene in human ovarian carcinomas is mediated via mitogen-activated kinase-dependent and -independent mechanisms. Int J Cancer 2005; 116: 894-902.
63. Tsai FM, Shyu RY, Jiang SY. RIG1 suppresses Ras activation and induces cellular apoptosis at the Golgi apparatus. Cell Signal 2007; 19: 989-999.
64. Xu F, Li X, Zhang P, Xia J, Wang Y, Yang C, et al. Melanoma differentiation-associated gene 5 is involved in the induction of stress granules and autophagy by protonophore CCCP. Biol Chem 2016; 397: 67-74.
65. Yang K, Wang J, Xiang AP, Zhan X, Wang Y, Wu M, et al. Functional RIG-I-like receptors control the survival of mesenchymal stem cells. Cell Death Dis 2013; 4: e967.
66. Molineros JE, Maiti AK, Sun C, Looger LL, Han S, Kim-Howard X, et al. Admixture mapping in lupus identifies multiple functional variants within IFIH1 associated with apoptosis, inflammation, and autoantibody production. PLoS Genet 2013; 9: e1003222.
67. Besch R, Poeck H, Hohenauer T, Senft D, Hacker G, Berking C, et al. Proapoptotic signaling induced by RIG-I and MDA-5 results in type I interferon-independent apoptosis in human melanoma cells. J Clin Invest 2009; 119: 2399-2411.
68. Lin L, Su Z, Lebedeva IV, Gupta P, Boukerche H, Rai T, et al. Activation of Ras/Raf protects cells from melanoma differentiation-associated gene-5-induced apoptosis. Cell Death Differ 2006; 13: 1982-1993.
69. Liu W, Wang X, Liu Z, Wang Y, Yin B, Yu P, et al. SGK1 inhibition induces autophagy-dependent apoptosis via the mTOR-Foxo3a pathway. Br J Cancer 2017; 117: 1139-1153.
70. Amato R, Menniti M, Agosti V, Boito R, Costa N, Bond HM, et al. IL-2 signals through Sgk1 and inhibits proliferation and apoptosis in kidney cancer cells. J Mol Med (Berl) 2007; 85: 707-721.
71. Chen Y, Azad MB, Gibson SB. Superoxide is the major reactive oxygen species regulating autophagy. Cell Death And Differentiation 2009; 16: 1040.
72. Chen Y, McMillan-Ward E, Kong J, Israels SJ, Gibson SB. Oxidative stress induces autophagic cell death independent of apoptosis in transformed and cancer cells. Cell Death Differ 2008; 15: 171-182.
73. Italiano D, Lena AM, Melino G, Candi E. Identification of NCF2/p67phox as a novel p53 target gene. Cell Cycle 2012; 11: 4589-4596.
74. Tey SK, Khanna R. Autophagy mediates transporter associated with antigen processing-independent presentation of viral epitopes through MHC class I pathway. Blood 2012; 120: 994-1004.
75. Neuzil J, Dong LF, Wang XF, Zingg JM. Tocopherol-associated protein-1 accelerates apoptosis induced by alpha-tocopheryl succinate in mesothelioma cells. Biochem Biophys Res Commun 2006; 343: 1113-1117.
76. Villarroya-Beltri C, Guerra S, Sanchez-Madrid F. ISGylation - a key to lock the cell gates for preventing the spread of threats. J Cell Sci 2017; 130: 2961-2969.
77. Nakashima H, Nguyen T, Goins WF, Chiocca EA. Interferon-stimulated gene 15 (ISG15) and ISG15-linked proteins can associate with members of the selective autophagic process, histone deacetylase 6 (HDAC6) and SQSTM1/p62. J Biol Chem 2015; 290: 1485-1495.
78. Zhou MJ, Chen FZ, Chen HC, Wan XX, Zhou X, Fang Q, et al. ISG15 inhibits cancer cell growth and promotes apoptosis. Int J Mol Med 2017; 39: 446-452.
79. Selleck EM, Fentress SJ, Beatty WL, Degrandi D, Pfeffer K, Virgin HWt, et al. Guanylate-binding protein 1 (Gbp1) contributes to cell-autonomous immunity against Toxoplasma gondii. PLoS Pathog 2013; 9: e1003320.
80. Smith EE, Malik HS. The apolipoprotein L family of programmed cell death and immunity genes rapidly evolved in primates at discrete sites of host-pathogen interactions. Genome Res 2009; 19: 850-858.
81. Bjorkoy G, Lamark T, Pankiv S, Overvatn A, Brech A, Johansen T. Monitoring autophagic degradation of p62/SQSTM1. Methods Enzymol 2009; 452: 181-197.
82. Chen S, Zhou L, Zhang Y, Leng Y, Pei XY, Lin H, et al. Targeting SQSTM1/p62 induces cargo loading failure and converts autophagy to apoptosis via NBK/Bik. Mol Cell Biol 2014; 34: 3435-3449.

83. Gomes AV. Genetics of proteasome diseases. Scientifica (Cairo) 2013; 2013: 637629.
84. Fullgrabe J, Ghislat G, Cho DH, Rubinsztein DC. Transcriptional regulation of mammalian autophagy at a glance. J Cell Sci 2016; 129: 3059-3066.
85. Xu-Monette ZY, Young KH. The TP53 tumor suppressor and autophagy in malignant lymphoma. Autophagy 2012; 8: 842-845.
86. Qi H, Xia FN, Xie LJ, Yu LJ, Chen QF, Zhuang XH, et al. TRAF Family Proteins Regulate Autophagy Dynamics by Modulating AUTOPHAGY PROTEIN6 Stability in Arabidopsis. Plant Cell 2017; 29: 890-911.
87. Speiser DE, Lee SY, Wong B, Arron J, Santana A, Kong YY, et al. A regulatory role for TRAF1 in antigen-induced apoptosis of T cells. J Exp Med 1997; 185: 1777-1783.
88. Ruckdeschel K, Mannel O, Schrottner P. Divergence of apoptosis-inducing and preventing signals in bacteria-faced macrophages through myeloid differentiation factor 88 and IL-1 receptor-associated kinase members. J Immunol 2002; 168: 4601-4611.
89. Ai Z, Lu Y, Qiu S, Fan Z. Overcoming cisplatin resistance of ovarian cancer cells by targeting HIF-1-regulated cancer metabolism. Cancer Lett 2016; 373: 36-44.
90. Sanchez-Martinez A, Calleja M, Peralta S, Matsushima Y, Hernandez-Sierra R, Whitworth AJ, et al. Modeling pathogenic mutations of human twinkle in Drosophila suggests an apoptosis role in response to mitochondrial defects. PLoS One 2012; 7: e43954.
91. Galavotti S, Bartesaghi S, Faccenda D, Shaked-Rabi M, Sanzone S, McEvoy A, et al. The autophagy-associated factors DRAM1 and p62 regulate cell migration and invasion in glioblastoma stem cells. Oncogene 2013; 32: 699-712.
92. Obba S, Hizir Z, Boyer L, Selimoglu-Buet D, Pfeifer A, Michel G, et al. The PRKAA1/AMPKalpha1 pathway triggers autophagy during CSF1-induced human monocyte differentiation and is a potential target in CMML. Autophagy 2015; 11: 1114-1129.
93. Azzam G, Wang X, Bell D, Murphy ME. CSF1 is a novel p53 target gene whose protein product functions in a feed-forward manner to suppress apoptosis and enhance p53-mediated growth arrest. PLoS One 2013; 8: e74297.
94. Lin X, Peng Z, Wang X, Zou J, Chen D, Chen Z, et al. Targeting autophagy potentiates antitumor activity of Met-TKIs against Met-amplified gastric cancer. Cell Death Dis 2019; 10: 139.
95. Xiao GH, Jeffers M, Bellacosa A, Mitsuuchi Y, Vande Woude GF, Testa JR. Anti-apoptotic signaling by hepatocyte growth factor/Met via the phosphatidylinositol 3-kinase/Akt and mitogen-activated protein kinase pathways. Proc Natl Acad Sci U S A 2001; 98: 247-252.
96. Wang G, Yang X, Li C, Cao X, Luo X, Hu J. PIK3R3 induces epithelial-to-mesenchymal transition and promotes metastasis in colorectal cancer. Mol Cancer Ther 2014; 13: 1837-1847.
